# Protection Against Persistent HPV-16/18 Infection After Different Number of Doses of Quadrivalent Vaccine in Girls and Young Women: A Randomized Clinical Trial

**DOI:** 10.1001/jamanetworkopen.2025.19095

**Published:** 2025-07-08

**Authors:** Chantal Sauvageau, Marie-Hélène Mayrand, Manale Ouakki, Iulia Gabriela Ionescu, François Coutlée, Julie Lacaille, Mélanie Benoit, Vladimir Gilca

**Affiliations:** 1 Centre de Recherche du Centre Hospitalier Universitaire de Québec-Université Laval, Québec, Québec, Canada; 2 Institut National de Santé Publique du Québec, Québec, Québec, Canada; 3 Social and Preventive Medicine Department, Université Laval, Québec, Québec, Canada; 4 Centre de Recherche du Centre Hospitalier de l’Université de Montréal, Montréal, Québec, Canada; 5 Obstetrics and Gynecology Department, Université de Montréal, Montréal, Québec, Canada; 6 Microbiology, Infectiology et Immunology Department, Université de Montréal, Montréal, Québec, Canada; 7 Molecular Diagnostic and Medical Microbiology Services, Clinical Department of Medical Laboratories, Centre Hopitalier de l’Université de Montréal, Montréal, Québec, Canada

## Abstract

**Importance:**

There are no randomized studies comparing the long-term effectiveness of a 2-dose schedule (administered at 0 and 6 months) of quadrivalent human papillomavirus vaccine (4vHPV) with a 2 + 1–dose schedule (administered at 0, 6, and 60 months) given to preadolescents for the prevention of persistent HPV-16 and HPV-18 infection.

**Objective:**

To evaluate whether a 2-dose 4vHPV schedule is noninferior to a 2 + 1–dose schedule for preventing persistent HPV-16 and HPV-18 infection up to 13 years after the first dose.

**Design, Setting, and Participants:**

This 2-arm, parallel, noninferiority, randomized clinical trial (RCT), called ICI-VPH (Impact of HPV Immunization Schedules Against HPV), was conducted in Québec, Canada, from 2013 to 2021. Eligible participants were girls who had received 2 doses of 4vHPV, 6 months apart when they were aged 9 to 11 years, 5 years prior to trial recruitment. Efficacy-evaluable population analysis was conducted between April 2023 and June 2024.

**Interventions:**

Participants were randomized 1:1 to the 2-dose or 2 + 1–dose groups. The 2 + 1–dose group received a booster dose of 4vHPV at the recruitment visit. Both groups were instructed on how to self-collect a vaginal sample, provided an initial vaginal swab, and completed an online questionnaire.

**Main Outcomes and Measures:**

The primary outcome was persistent HPV-16 and HPV-18 infection, defined as the presence of HPV-16 or HPV-18 DNA in 2 consecutive vaginal samples self-collected 5 to 15 months apart.

**Results:**

Among the 3356 participants, the mean (SD) age at enrollment was 14.8 (0.4) years, and 2867 (85.4%) were French Canadian individuals. Of the 3364 participants initially randomized, 8 (0.2%) withdrew immediately after randomization, 1675 (49.8%) were assigned to the 2-dose group, and 1681 (50.0%) were assigned to the 2 + 1–dose group. Of the 16 989 genotyping tests analyzed, 31 (0.2%) detected vaccine-type HPV-6, -11, -16, and -18. Only 1 participant in the 2 + 1–dose group (0.1%) had a time-limited persistent HPV-16 infection.

**Conclusions and Relevance:**

This clinical trial found that the incidence of HPV-16 and HPV-18 infection was low with both the 2-dose and 2 + 1–dose 4vHPV schedules. A 2-dose schedule provided robust protection against persistent HPV-16 and HPV-18 infection for up to 13 years, suggesting that a booster dose administered at 60 months would not provide additional benefit.

**Trial registration:**

ClinicalTrials.gov Identifier: NCT02009800

## Key Points

**Question:**

Is a 2-dose schedule (administered at 0 and 6 months) of quadrivalent human papillomavirus vaccine (4vHPV) noninferior to a 2 + 1–dose schedule (administered at 0, 6, and 60 months) for preventing persistent HPV-16 and HPV-18 infection up to 13 years after the first dose?

**Findings:**

In this randomized clinical trial of 3356 participants, both 2-dose and 2 + 1–dose schedules resulted in few HPV-16 and HPV-18 infections. Two doses provided robust protection against persistent HPV-16 and HPV-18 infection for up to 13 years.

**Meaning:**

The findings suggest that a booster dose administered at 60 months would not provide additional benefit.

## Teaser

This randomized clinical trial evaluates the long-term protection from 2 doses vs 2 doses with a booster of the quadrivalent human papillomavirus vaccine.

## Introduction

Human papillomaviruses (HPVs) are responsible for most anogenital and oropharyngeal cancers and anogenital warts.^1^ Safe and highly protective HPV vaccines have been available for nearly 2 decades.^1,2^ These vaccines were initially licensed for use in females aged 9 to 26 years using a 3-dose, 6-month schedule based on immunogenicity and efficacy data from randomized clinical trials (RCTs) conducted in females aged 16 to 26 years.^3,4^ Since then, several studies have investigated alternative schedules with a lower number of doses and/or longer interval between doses. A schedule of particular interest is the administration of a booster dose just before the age when many adolescents begin sexual activity, as this would ensure the highest antibody levels at the time of greatest risk of infection. This is also a time when adolescents can be easily reached (ie, before leaving school) and when other vaccines are administered as part of an immunization program, thus optimizing the use of existing resources.^5^

In 2008, the province of Québec, Canada, introduced its school-based vaccination program with the quadrivalent HPV vaccine (4vHPV), recommending an extended schedule with the first 2 doses administered 6 months apart in grade 4 and a third dose, if needed, in grade 9 (ie, 60 months after the first dose).^6^ In 2013, the Québec Immunisation Committee recommended not administering the third dose and evaluating the comparative efficacy and immunogenicity of the 2 schedules.^7^ No randomized study has compared the effectiveness of the 2-dose (administered at 0 and 6 months) and 2 + 1–dose (administered at 0, 6, and 60 months) schedules for up to 13 years.

The primary objective of the ICI-VPH (Impact of HPV Immunization Schedules Against HPV) trial was to evaluate whether a 2-dose 4vHPV schedule is noninferior to a 2 + 1–dose schedule for preventing persistent HPV-16 and HPV-18 infection up to 10 years after the first dose. Follow-up was then extended up to 13 years.

## Methods

### Trial Design and Participants

The ICI-VPH is a 2-arm, parallel, noninferiority RCT with single masking. The Research Ethics Committees of the Centre Hospitalier Universitaire de Québec-Université Laval, the Centre Hospitalier de l’Université de Montréal, and the Centre Intégré Universitaire de Santé et de Services Sociaux du Saguenay–Lac-Saint-Jean approved the study. The full protocol is provided in Supplement 1. Written informed consent was obtained from both participants and their parents. We followed the Consolidated Standards of Reporting Trials (CONSORT) reporting guideline.

Participants were recruited from 3 health regions in Québec, Canada. Each year between 2013 and 2016, we contacted by mail and/or telephone the parents of girls who were in grade 4 (aged 9-11 years) 5 years earlier, when they received the first dose of 4vHPV as part of Québec’s HPV vaccination program. Eligibility required girls to have received 2 doses of 4vHPV 6 months apart (minimum of 4 to maximum of 12 months), immunocompetence at administration of first dose and at recruitment, not currently pregnant, and residency in one of the recruitment areas. Finally, parents and participants needed to understand French or English.

Parents’ contact information was obtained through the Régie de l’Assurance Maladie du Québec after approval by the Comission d’Accès à l’Information du Québec. Interested parents and their daughters were scheduled for an appointment at the regional research center, where eligibility was confirmed. Pregnancy was determined using urine β-human chorionic gonadotropin testing.

### Randomization and Intervention

Participants were randomized 1:1 to either the 2-dose or 2 + 1–dose group, according to computer-generated random treatment allocation lists using variable-permuted block sizes of 10, 12, and 14, with a separate list for each research center. An independent statistician generated the lists, which were uploaded into the REDCap (Research Electronic Data Capture; Vanderbilt University) randomization module. The 2 + 1–dose group received a 4vHPV booster dose at recruitment (ie, 60 months after the first dose). Given that the trial was single-masked, only study personnel (F.C. and M.O.) assessing outcomes (HPV DNA) were blinded to group allocation.

### Study Procedures

**Figure 1.**
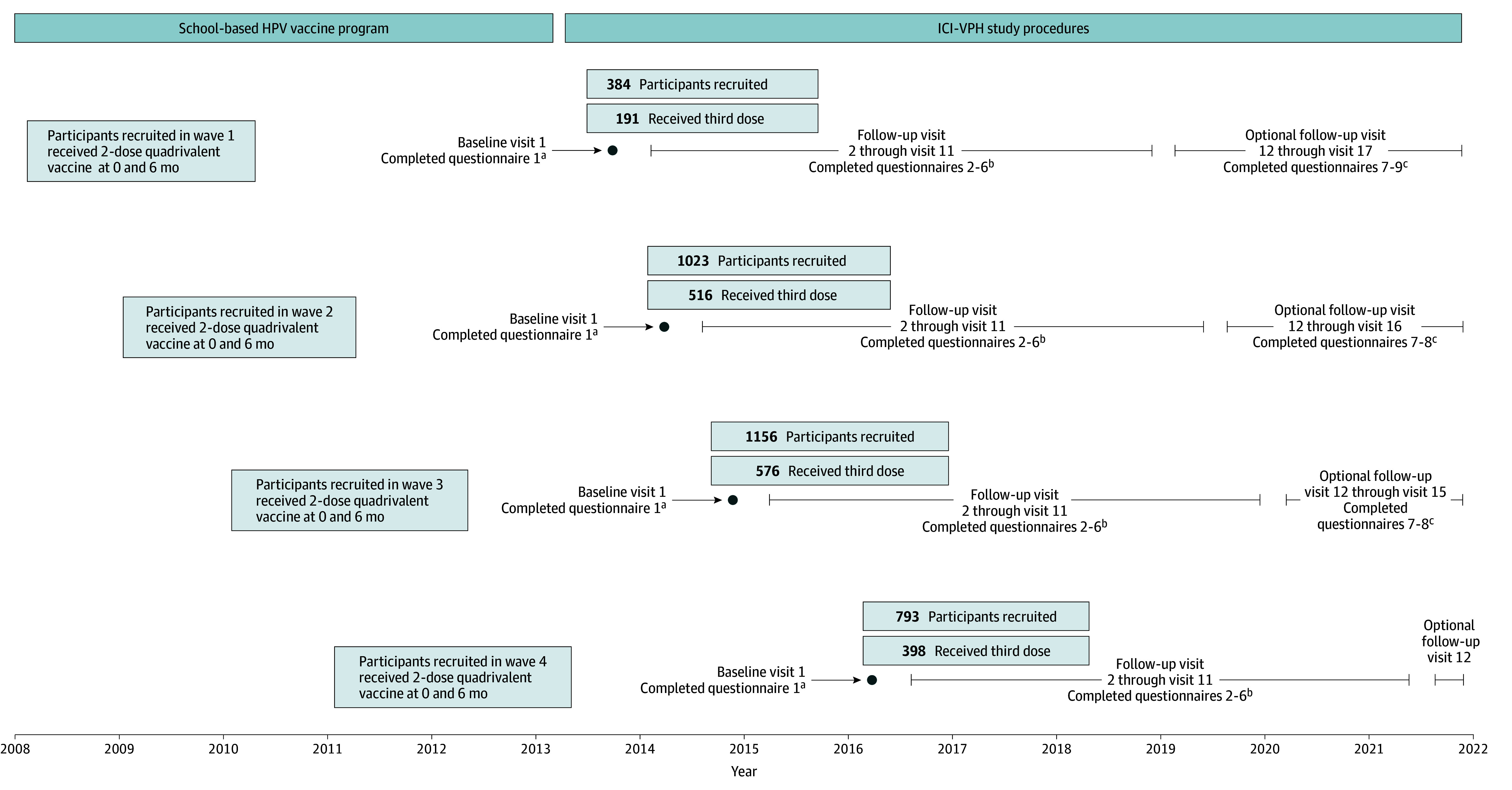
Impact of HPV Immunization Schedules Against HPV (ICI-VPH) Study Procedures HPV indicates human papillomavirus. ^a^Participants provided the initial vaginal swabs and were instructed on how to self-collect vaginal samples. ^b^Participants started self-sampling every 6 months from visit 2 through visit 11. Study staff tested samples at even-numbered time points (eg, visit 2, visit 4). If a sample tested positive for HPV, previous and subsequent swabs were tested. Participants completed yearly questionnaires at odd-numbered time points (eg, visit 3, visit 5). ^c^Some participants continued follow-up and completed questionnaires at odd-numbered time points.

At the recruitment visit (visit 1), participants were instructed on how to self-collect a vaginal sample (self-sampling) and were provided with an initial vaginal swab. They also completed an online questionnaire collecting demographic data (eg, age, ethnicity), medical history, and sexual and reproductive behaviors (Figure 1). Ethnicity data were collected and analyzed in this trial to describe the study population and assess its representativeness compared with the population of adolescent girls in Québec. Self-reported ethnicities included English Canadian; French Canadian; Latin American, South American, or Central American; or other (Aboriginal/First Nations, Black [Africa], Black [West Indies, US, and South America], British, East Asian [eg, Chinese, Japanese, and Korean], French [France], Greek, Italian, Jewish of European descent, Jewish of Sephardic descent, North or Middle Eastern [eg, Afghan, Iranian, and Arab], Other European [eg, German, Dutch, and Ukrainian], Portuguese, Scottish or Irish, South Asian [eg, East Indian, Pakistani, and Sri Lankan], Southeast Asian [eg, Cambodian, Filipino, Indonesian, and Vietnamese], and other or unspecified ethnicity).

Participants received vaginal self-sampling kits by mail every 6 months for 5 years (visit 2 through visit 11) except for a 6-month interruption (March 23 to October 4, 2020) due to the COVID-19 pandemic. A web link to a follow-up questionnaire was sent once a year to solicit updates on lifestyle and reproductive and sexual health. Because follow-up for the last recruited participants ended in 2021, all participants were given the option to continue their follow-up until that time (ie, up to 13 years after the first dose for the first recruited participants).

### Outcomes

The primary outcome was persistent HPV-16 and HPV-18 infection, defined as the presence of either HPV-16 or HPV-18 DNA in 2 consecutive vaginal samples self-collected 5 to 15 months apart. Persistent infection is the preferred surrogate outcome to evaluate vaccine effectiveness in a timely manner.^8,9^ Secondary outcomes were geometric mean antibody concentrations and seropositivity proportions of HPV-6, -11, -16, -18, -31, -33, -45, -52, and -58 between the 2 study groups at 60, 90, and 120 months after the first dose; these outcomes are the subject of a separate investigation.

We originally planned to test all vaginal swabs for HPV DNA. However, after recruiting approximately half of the target sample, we found that the low prevalence of sexual activity among 14- to 15-year-old participants resulted in few HPV detections; thus, testing all swabs was considered an inefficient resource use. Because the primary outcome was persistent infection, we stopped routinely testing samples from baseline (visit 1) and other odd-numbered time points (eg, visit 3, visit 5). Participants continued to collect samples every 6 months, but we tested only samples from even-numbered time points (eg, visit 2, visit 4). When a sample tested positive for HPV, we tested the previous and subsequent swabs.

Swabs were suspended in a buffer of Tris 10mM plus EDTA 0.1mM at pH 8.3 and lysed with the addition of proteinase K and Tween 20 at a final concentration of 0.7 μg/μL and 0.8%, respectively, at 65 °C for 1.5 hours and then at 95 °C for 10 minutes. Cell lysates were first tested for the presence of HPV DNA using a generic HPV assay.^10^ HPV-negative lysates using the generic assay were further analyzed for the presence of β-globin DNA sequence by polymerase chain reaction (PCR).^11^ β-Globin–negative samples were further processed using the purification kit MasterPure (Biosearch Technologies Inc).^12^ The purified DNA was then tested for β-globin; if positive for β-globin, the DNA was tested using the same generic HPV assay. Samples that were positive for β-globin and negative for HPV were considered negative for HPV. β-Globin–negative samples after the MasterPure processing were considered inadequate for PCR analysis.

Genotyping of HPV-positive samples was performed using the Linear Array (Roche Molecular Systems), which identified 36 HPV genotypes,^13^ until discontinuation in 2020. Next, we used the Anyplex II HPV28 Detection assay (Seegene Inc), which identified 28 genital genotypes.^14^ Samples reactive for HPV-52 in the Linear Array were then analyzed using a validated HPV-52–specific real-time PCR assay.^12^

HPV risk categories were based on the International Agency for Research on Cancer classification, with HPV genotypes 16, 18, 31, 33, 35, 39, 45, 51, 52, 56, 58, 59, and 68 considered to be high risk.^15^ The 4vHPV targets HPV genotypes 6, 11, 16, and 18.

### Sample Size Calculation

To assess the noninferiority of a 2-dose schedule over a 2 + 1–dose 4vHPV schedule, we determined sample size based on the cumulative incidence of persistent HPV infections reported in the literature^16,17^ and the expected effectiveness of the 2-dose and 2 + 1–dose schedules (94% and 95% effectiveness, respectively).^18,19,20^ With 90% power (as recommended^21^), α = .05, and a 5% noninferiority margin, the required sample size was 3334 participants (1667 per group), accounting for 5% annual loss to follow-up over 5 years.

### Statistical Analysis

The number of HPV-positive samples at even-numbered time points was compared between study groups using χ^2^ or Fisher exact test. Statistical significance was set at a 2-sided *P* = .05, and Bonferroni correction was used for multiple comparisons. The proportions of HPV-positive samples and 95% CIs were graphically presented by study group over the initially planned 5 years of follow-up and the optional 3-year extension period.

We originally planned to perform a noninferiority analysis of vaccine schedules and persistent HPV-16 and HPV-18 infection. However, due to higher-than-expected vaccine effectiveness in both groups and an insufficient number of events, this analysis could not be performed and thus noninferiority was not assessed. All statistical analyses followed the efficacy-evaluable population approach and were performed using SAS, version 9.4 (SAS Institute Inc), between April 2023 and June 2024.

## Results

### Trial Population

A total of 3356 girls and young women were included in the analysis. Baseline characteristics were similar between groups (Table 1). Participants had a mean (SD) age at recruitment of 14.8 (0.4) years; were predominantly born in Canada (3094 [92.2%]); and self-reported their ethnicities (more than 1 possible) as follows: 190 English Canadians (5.7%), 2867 French Canadians (85.4%), 140 Latin Americans, South Americans, or Central Americans (4.2%), and 924 other ethnicities (27.5%). Among participants, 711 (21.2%) reported using hormonal contraception, 264 (7.9%) reported ever having smoked a cigarette, and 539 (16.1%) reported ever having had sex. Of those who reported having had penetrative sex, 292 (67.3%) used a condom during their last sexual intercourse.

**Table 1.** Baseline Characteristics of Participants

Characteristics	Participants, No. (%)		
	2-Dose group (n = 1675)	2 + 1–Dose group (n = 1681)	Total (n = 3356)
Age at recruitment, mean (SD), y	14.8 (0.4)	14.8 (0.4)	14.8 (0.4)
Ethnicity^a^			
English Canadian	110 (6.6)	80 (4.8)	190 (5.7)
French Canadian	1429 (85.3)	1438 (85.5)	2867 (85.4)
Latin American, South American, or Central American	76 (4.5)	64 (3.8)	140 (4.2)
Other^b^	487 (29.1)	437 (26.0)	924 (27.5)
Country of birth			
Canada	1552 (92.7)	1542 (91.7)	3094 (92.2)
Other^c^	123 (7.3)	139 (8.3)	262 (7.8)
Region of residence			
Montréal	847 (50.6)	849 (50.5)	1696 (50.5)
Québec	719 (42.9)	722 (43.0)	1441 (42.9)
Saguenay–Lac-Saint-Jean	109 (6.5)	110 (6.5)	219 (6.5)
Tobacco use			
Ever smoked 1 cigarette	136 (8.1)	128 (7.6)	264 (7.9)
Daily smoker	18 (1.1)	14 (0.8)	32 (1.0)
Sexual health			
Current user of hormonal contraception	359 (21.4)	352 (20.9)	711 (21.2)
Ever had sex	285 (17.0)	254 (15.1)	539 (16.1)
Ever had an STI^d^	6 (2.1)	2 (0.8)	8 (1.5)
Ever had penetrative sex^d^	233 (81.8)	201 (79.1)	434 (80.5)
Used condom during past intercourse^e^	158 (67.8)	134 (66.7)	292 (67.3)

Abbreviation: STI, sexually transmitted infection.

^a^ Participants could report more than 1 ethnicity; thus, totals do not equal 100%.

^b^ Includes Aboriginal/First Nations, Black (Africa), Black (West Indies, US, and South America), British, East Asian (eg, Chinese, Japanese, and Korean), French (France), Greek, Italian, Jewish of European descent, Jewish of Sephardic descent, North or Middle Eastern (eg, Afghan, Iranian, and Arab), Other European (eg, German, Dutch, and Ukrainian), Portuguese, Scottish or Irish, South Asian (eg, East Indian, Pakistani, and Sri Lankan), Southeast Asian (eg, Cambodian, Filipino, Indonesian, and Vietnamese), and other or unspecified ethnicity.

^c^ Participants who were born outside of Canada were not asked to specify their country of birth.

^d^ Denominator includes only those who ever had sex.

^e^ Denominator includes only those who ever had penetrative sex.

**Figure 2.**
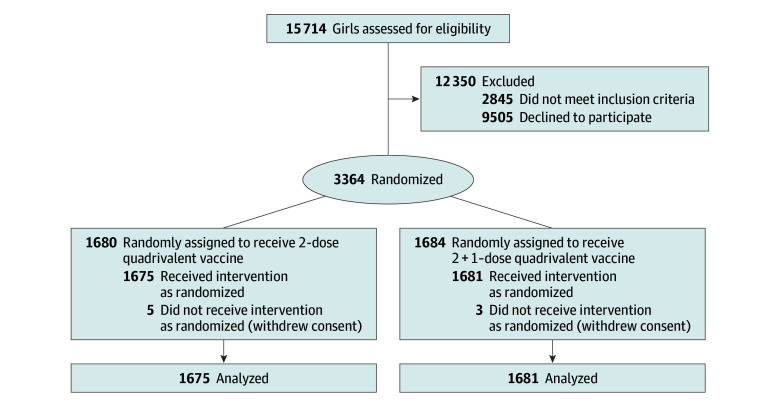
CONSORT Flow Diagram

A total of 73 307 recruitment letters were sent out in 4 waves, each corresponding to a school year from 2013 to 2016. Additionally, 15 714 parents were contacted by telephone to assess the eligibility of their daughters. Of the 12 869 eligible girls and young women, 3364 (26.1%) consented with their parents to participate in the study. Eight participants (0.2%) withdrew immediately after randomization, leaving 1675 (49.8%) and 1681 (50.0%) participants in the 2-dose and 2 + 1–dose groups, respectively (Figure 2).

The median (IQR) follow-up after the first vaccine dose was 10.9 (10.0-11.9) years, with a maximum of 13.3 years. At the final completed study questionnaire, participants ranged in age from 14 to 23 years, with a mean (SD) age of 20.2 (1.3) years. At least once during follow-up, 2733 participants (81.4%) reported using hormonal contraception and 2780 (82.8%) reported being sexually active, of whom 413 (14.9%) reported ever having had a sexually transmitted infection (eTable 1 in Supplement 2).

### HPV Infections

Participants sent a total of 36 371 vaginal samples (18 170 from those in the 2-dose group; 18 201 from those in the 2 + 1–dose group), with 2122 participants (63.2%) sending all scheduled swabs, while 376 (11.2%) missed 3 or more returns. At even-numbered visits 2 through 10, we found that 988 (6.4%) of 15 508 swabs were not returned by participants (eTable 3 in Supplement 2). Of the returned swabs, 257 (1.8%) were invalid and did not produce an HPV result.

**Figure 3.**
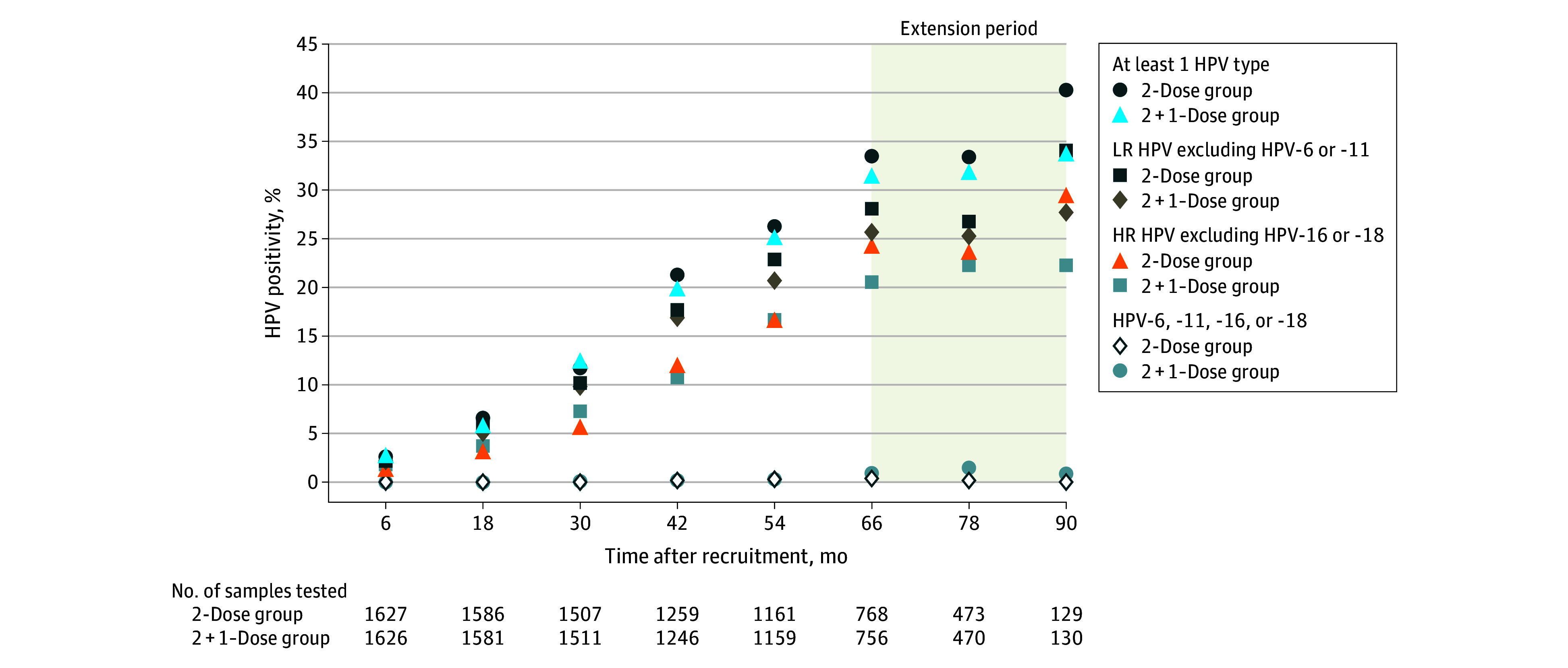
Human Papillomavirus (HPV) Positivity in Participants With 2-Dose vs 2 + 1–Dose Quadrivalent Vaccine Schedule at Even-Numbered Time Points During Follow-Up The extension period was optional, with some participants continuing follow-up for an additional 3 years. HR indicates high risk and LR indicates low risk.

Of the adequate swabs collected at even-numbered time points, 1573 participants (18.5%) in the 2-dose group and 1527 participants (18.0%) in the 2 + 1–dose group had an HPV-positive result (generic test), with 2663 (85.9%) confirmed by genotyping. A total of 2023 participants (60.3%) (1008 [60.2%] and 1015 [60.4%] in the 2-dose and 2 + 1–dose groups, respectively) had no positive HPV test results throughout the study period. For all genotypes, HPV positivity increased during follow-up (Table 2, Figure 3), indicating that more infections occurred with increasing age and sexual activity. The incidence of vaccine-type HPVs (genotypes 6, 11, 16, and 18) remained low (<1.0%) at each even-numbered time point in both study groups. Of the 16 989 genotyping tests performed, 31 (0.2%) in 28 participants identified HPV genotypes 6, 11, 16, or 18 (eTable 2 in Supplement 2). There were 8 (0.1%) HPV-16 and HPV-18 types detected in the 2-dose group and 15 (0.2%) in the 2 + 1–dose group. There was 1 (<0.1%) HPV-6 or -11 type in the 2-dose group and 7 (0.1%) in the 2 + 1–dose group. Differences were not significant (*P* > .05).

**Table 2.** Human Papillomavirus (HPV) Positivity in Self-Collected Samples at Even-Numbered Time Points^a^

	2-Dose group								2 + 1–Dose group							
Time since recruitment, mo	6	18	30	42	54	66	78	90	6	18	30	42	54	66	78	90
Time since first dose, y	6	7	8	9	10	11	12	13	6	7	8	9	10	11	12	13
No. of samples (tested and valid)	1627	1586	1507	1259	1161	768	473	129	1626	1581	1511	1246	1159	756	470	130
HPV-positive, No. (%)	43 (2.6)	105 (6.6)	176 (11.7)	268 (21.3)	305 (26.3)	257 (33.5)	158 (33.4)	52 (40.3)	45 (2.8)	93 (5.9)	189 (12.5)	248 (19.9)	292 (25.2)	238 (31.5)	150 (31.9)	44 (33.8)
At least 1 HR HPV	22 (1.4)	51 (3.2)	86 (5.7)	154 (12.2)	196 (16.9)	190 (24.7)	113 (23.9)	38 (29.5)	29 (1.8)	56 (3.5)	111 (7.3)	135 (10.8)	196 (16.9)	161 (21.3)	107 (22.8)	30 (23.1)
Single infection	23 (1.4)	45 (2.8)	72 (4.8)	98 (7.8)	99 (8.5)	69 (9.0)	51 (10.8)	16 (12.4)	18 (1.1)	35 (2.2)	84 (5.6)	89 (7.1)	103 (8.9)	73 (9.7)	51 (10.9)	11 (8.5)
Multiple infections	20 (1.2)	60 (3.8)	104 (6.9)	170 (13.5)	206 (17.7)	188 (24.5)	107 (22.6)	36 (27.9)	27 (1.7)	58 (3.7)	105 (6.9)	159 (12.8)	189 (16.3)	165 (21.8)	99 (21.1)	33 (25.4)
LR HPV excluding HPV-6 or -11	35 (2.2)	95 (6.0)	154 (10.2)	223 (17.7)	266 (22.9)	216 (28.1)	127 (26.8)	44 (34.1)	35 (2.2)	77 (4.9)	148 (9.8)	210 (16.9)	240 (20.7)	194 (25.7)	119 (25.3)	36 (27.7)
HR HPV excluding HPV-16 or -18	22 (1.4)	51 (3.2)	86 (5.7)	152 (12.1)	194 (16.7)	187 (24.3)	112 (23.7)	38 (29.5)	29 (1.8)	56 (3.5)	110 (7.3)	134 (10.8)	193 (16.7)	156 (20.6)	105 (22.3)	29 (22.3)
HPV-6 or -11	0	0	0	0	1 (0.1)	0	0	0	0	0	0	1 (0.1)	0	2 (0.3)	4 (0.9)	0
HPV-16 or -18	0	0	0	2 (0.2)	2 (0.2)	3 (0.4)	1 (0.2)	0	0	0	2 (0.1)	1 (0.1)	3 (0.3)	5 (0.7)	3 (0.6)	1 (0.8)

Abbreviations: HR, high risk; LR, low risk.

^a^ Between-group comparisons were made at even-numbered time points using χ^2^ or Fisher exact test, with Bonferroni correction for multiple comparisons. All *P* ≥ .05.

Only 1 participant (0.1%) in the 2 + 1–dose group had a confirmed time-limited persistent HPV-16 infection (ie, 2 positive samples 12 months apart, followed by 2 HPV-16–negative samples). Five participants (0.2%)—3 in the 2-dose group, and 2 in the 2 + 1–dose group—had HPV-16 and HPV-18 types detected in their last sample, making it impossible to rule out persistence. The incidence of nonvaccine HPV genotypes at even-numbered time points varied from 0 to 3.5%. The most common high-risk HPV genotypes were 51, 59, 39, 52, 56, and 58.

## Discussion

In this cohort of 3356 young women vaccinated in grade 4 (aged 9-11 years) as part of the Québec HPV vaccination program, few HPV infections (genotypes 6, 11, 16, and 18) were detected at recruitment (grade 9, 5 years after initial vaccination) and during follow-up, demonstrating the lasting effectiveness of HPV vaccination up to 13 years after the first dose. There were 23 incident HPV-16 and HPV-18 infections, 1 time-limited persistent HPV-16 and HPV-18 infection, and 5 HPV-16 and HPV-18 infections at the last study sample. The low number of persistent infection did not allow for a noninferiority analysis, but it highlights the immense success of the Québec HPV vaccination program. Persistent high-risk HPV-16 and HPV-18 infection, a recognized indicator of vaccine efficacy,^8,9^ is estimated to be responsible for 70% of cervical cancers.^22^ Therefore, our results foreshadow a drastic decrease in cervical cancer in the coming years.^23^

### Results in the Context of What Is Known

The low incidence of HPV-16 and HPV-18 infection in this study reflects the high effectiveness of a 2-dose schedule, with similar results reported in nonrandomized studies of young, vaccinated females.^24,25,26,27,28,29^ For instance, an Indian study showed a low incidence (0.1%), similar to the present results, of persistent HPV-16 and HPV-18 infection over 12 years of follow-up after administration of 1, 2, or 3 doses of 4vHPV to girls aged 10 to 18 years.^25^ However, most studies on the efficacy or effectiveness of a 2-dose schedule in preventing HPV-16 and HPV-18 infection focused on women aged 18 years or older,^24,26,27,28,29^ missing the adolescent period when HPV exposure risk is high due to initiation of sexual activity and, in many cases, multiple sexual partners.^30^ Additionally, few studies evaluated long-term (>10 years) effectiveness.^25^

Two RCTs^31,32^ evaluated the impact of a 4vHPV booster dose (given at 42 or 50 months) following a 2-dose schedule but reported only immunogenicity outcomes. They showed noninferiority of extended schedules compared with doses given in a 6-month interval.^31,32^ Consistent with these findings, our results suggest that a booster dose at 60 months may not provide additional benefit against persistent HPV-16 and HPV-18 infection for up to 13 years after the first dose, given the near-complete protection provided by the 2-dose, 6-month schedule.

Furthermore, we observed only 8 incident HPV-6 and −11 infections. Two meta-analyses have shown that the 4vHPV can prevent genital warts in girls and women.^33,34^ Although we did not assess the presence of condylomas among participants, the low number of HPV-6 and −11 infections suggests excellent protection against condylomas, the majority (90%) of which are associated with HPV genotypes 6 and 11.^35^ Two of the most common high-risk HPV genotypes—52 and 58—found in the ICI-VPH trial are not covered by the 4vHPV but are targeted by the nonavalent HPV vaccine (9vHPV) now used in Canada.

### Implications for Practice and Future Research

The Québec Immunization Committee and the National Advisory Committee on Immunization recently recommended the use of a 1-dose schedule,^36,37^ as recent data suggest a single dose offers high efficacy similar to 2- or 3-dose schedules in preventing persistent HPV-16 and HPV-18 infection for up to 12 years after vaccination.^25,38,39^ Findings of the present study suggest that a booster dose administered at 60 months provides no additional benefit to a 2-dose schedule and, therefore, may provide no additional benefit to a 1-dose schedule. Given the unknown duration of protection provided by HPV vaccines, the impact of a booster dose administered later than 5 years after primary vaccination warrants further investigation.

All study participants received the 4vHPV, which has been replaced by the 9vHPV in Québec (2016) and in several jurisdictions since the start of the ICI-VPH trial. However, the 4vHPV and 9vHPV vaccines are highly comparable using the same manufacturing process and adjuvant and both protect against the 4 HPV types (6, 11, 16, and 18) responsible for most HPV-associated cancers and condylomas.^3,40^ The 9vHPV shows a similar antibody response and efficacy against persistent HPV-16 and HPV-18 infection compared with the 4vHPV,^38,40,41^ making our 4vHPV-based observations useful for understanding the performance of 9vHPV and estimating its protection duration given the longer use of the 4vHPV vaccine.

### Strengths and Limitations

Key strengths of the ICI-VPH trial include its randomized design, large sample size, up to 13 years of follow-up, and high adherence to study procedures. All participants (aged 9-11 years) were initially vaccinated as part of the school-based Québec HPV vaccination program, allowing for assessment in a public health context, with vaccination (2 doses) occurring when exposure to HPV was low, and virtually eliminating any potential effect of prevaccination natural infection on vaccine-induced protection.

Among the limitations is that this trial was partially blinded, as the 2-dose group did not receive a placebo. The administration of a placebo had potential drawbacks (eg, adverse events, cost, and resource requirements) and was not considered ethically acceptable or scientifically necessary. Laboratory analyses were blinded to minimize potential bias in assessing persistent infection.

The low level of participation may raise concerns about sample representativeness, but the distribution of several characteristics, such as smoking, sexual initiation, condom use, and sexually transmitted infections, suggests that the sample is representative of the population of adolescents and young women in Québec.^30,42,43^ Although it was challenging to include participants from nonmajority sociocultural groups despite study procedures that ensured contact by letter and/or telephone with all eligible girls, previous multicenter studies have shown no differences in vaccine efficacy across ethnicities.^3,4,16,18,40^

Because only 1 persistent HPV-16 infection was detected, the noninferiority analysis could not be completed. Nevertheless, the descriptive results alone reflect excellent effectiveness of this public health program and the expected impact on the health of the Québec population and other populations with similar immunization programs. The COVID-19 pandemic led to a 6-month study halt during which no self-sampling kits were sent to participants.

## Conclusions

In this RCT of 3356 girls, 2 doses of 4vHPV administered 6 months apart provided near-complete protection against persistent HPV-16 and HPV-18 infection for up to 13 years after the first dose. This finding suggests that a booster dose administered 5 years after the first 2 doses may not provide additional benefit. Nevertheless, HPV vaccination is an exceptionally effective preventive intervention.

## Notes

Supplement 1.
Trial Protocol

Supplement 2. **eTable 1.** Characteristics of Study Participants in the Annual Follow-Up Questionnaires **eTable 2.** HPV Positivity at Even-Numbered Time Points According to Vaccine Schedules **eTable 3.** Number of Swab Kits Sent, Received, and Tested at Each Even-Numbered Time Point for Up to 10 Years After the First Dose

Supplement 3.
Data Sharing Statement
