# Supplementary material for: Protection Against Persistent HPV-16/18 Infection After Different Number of Doses of Quadrivalent Vaccine in Girls and Young Women: A Randomized Clinical Trial
Source: JAMA Netw Open. 2025 Jul 8;8(7):e2519095. doi: 10.1001/jamanetworkopen.2025.19095 (PMC12238905; doi:10.1001/jamanetworkopen.2025.19095)

PROTOCOL

ICI-VPH : Impact des calendriers d'immunisation contre les VPH

Principal Investigators : Chantal Sauvageau

Marie-Hélène Mayrand

Co-investigators : Vladimir Gilca

François Coutlée

Marc Dionne

Nicole Boulianne

Collaborators : Christian Therrien

Manale Ouakki

Gitika Panicker

Elizabeth R. Unger

Financial support : Ministère de la Santé et des Services sociaux du Québec

Michael Smith Foundation

Version #13.1 - Non-official English translation of the original French version approved by the REB

August 2022

Confidential

TABLE OF CONTENT

1. WHY A STUDY ON HPV VACCINE SCHEDULES IN THE PROVINCE OF QUEBEC? 3
- 1.1 HPV and Associated Diseases: General Epidemiological Considerations ... 3
- 1.2 Prevention of cervical cancer: screening and vaccination 3
- 1.3 HPV vaccination in Canada and in Quebec 4
- 1.4 HPV vaccination: benefit of alternative schedules 4
- 1.5 Why is it essential to conduct a study on HPV vaccine schedules in Quebec now? 5
- 1.6 Overlap between ICI-VPH and the Canadian study (QUEST) comparing a 2-dose schedule (0, 6 months) to a 3-dose schedule (0, 2, 6 months). 6

2. OBJECTIVES 7
- 2.1 Primary objective 7
- 2.2 Secondary objectives 7

3. PROPOSED STUDY 7
- 3.1 Study design 7
- 3.2 Study population 7
- 3.3 Enrolment 8
- 3.4 Conduct of the study 8
- 3.5 Study variables and effect measures 9
  - 3.5.1 Intervention: definition and measurement 9
  - 3.5.2 Primary outcome: definition and measurement 9
  - 3.5.3 Secondary outcomes: definition and measurement 10
  - 3.5.4 Sample preservation and storage 11
- 3.6 Randomization 12
- 3.7 Bias protection 12
- 3.8 Sample size and feasibility of enrolment and follow-up 12
  - 3.8.1 Sample size needed to answer the primary research question. 12
  - 3.8.2 Sample size for the immunogenicity section 17
  - 3.8.3 Statistical power for the objectives on the impact on condylomas 17
  - 3.8.4 Feasibility of enrolment and follow-up. 18
- 3.9 Analysis 18

4. STUDY MANAGEMENT 19
- 4.1 TEAM 19
- 4.2 Coordination 20
- 4.3 Calendar 20

5. ETHICS CONSIDERATIONS 21
- 5.1 Confidentiality 21
- 5.2 External committee 21

6. EXPECTED IMPACT 22

7. EXTENSION : PHASE 2 22

REFERENCES 23

APPENDIX A – SUMMARY OF THE CANADIAN STUDY QUEST: QUADRIVALENT HPV VACCINE EVALUATION STUDY 33

APPENDIX B – CONDUCT OF THE STUDY 36

1. Why a study on HPV vaccine schedules in the province of Quebec?

1.1 HPV and Associated Diseases: General Epidemiological Considerations

Human papilloma viruses (HPV) infect the skin and mucosae. There are over a hundred types of HPVs, which are identified by numbers(1,2). About forty types of human papilloma viruses can infect the anogenital mucosa. Between 15 and 25(3) of these viruses have been associated with the development of cancerous or precancerous lesions, and as a result, these viruses are known as oncogenic or high-risk viruses(3). HPVs have been associated with cancers of the: cervix, vagina, vulva, anus, penis and certain cancers in the oro-pharyngyal region. HPV types 16 and 18 are respectively the 1st and 2nd viruses most frequently identified in these cancers(4). Together, HPV types 16 and 18 appear to be responsible for about 70% of cervical cancers worldwide(4).

Infection of the genital mucosae by one HPV is very frequent. The lifetime risk of acquiring at least one HPV genital infection has been estimated at more than 70%(5). These infections usually occur in the years following onset of sexual activity(6,7). Transmission mainly results from intimate and prolonged contact with infected skin/mucosa. Most infected persons will clear the infection without sequelae (even those infected with oncogenic viruses)(8). However in a few, infection will persist and might eventually trigger precancerous or cancerous modifications(9–11). Because of its histologic features, the mucosa of the cervix is particularly vulnerable to the oncogenic effect of high risk viruses.

Between 2004 and 2007 in Quebec, there were about 281 new cervical cancers and 69 deaths per year(12). It was also estimated that approximately 131 HPV-associated cancers occurred yearly at other anatomical sites (vulva, vagina, oropharynx…) in Quebec women(12).

1.2 Prevention of cervical cancer: screening and vaccination

Fortunately, precancerous modifications of the cervix evolve towards cancer slowly. The peak of age of new HPV infections occurs between 20 to 24 years of age, that of precancerous lesions at 25-35 years of age and invasive cancers at 50 years(13). Precancerous modifications can be identified by a cytological exam of samples collected at the cervix (Pap test). Women with abnormal Pap test result(s) will be directed to colposcopy for diagnosis. If a precancerous lesion is identified, its treatment will prevent progression to cancer.

Activities related to cervical cancer screening have been in place in Quebec for more than 50 years. These activities, which are opportunistic, had a certain impact on the incidence of and mortality from cervical cancer(13). However, this success is quite expensive given that screening have to begin in the twenties and be repeated every 2 to 3 years till the sixties(14). False positive results cause an important number of unnecessary exams. Additionally, some treatments of precancerous lesions have been associated with an increased risk of premature deliveries and neonatal mortality(15,16). Finally, nowadays, the main reason of cervical cancer related death is a non-use or insufficient use of screening by some women(17). These women mainly originate from the most vulnerable groups, thus raising a question of social equity. Some programs have implemented measures to reach these high risk groups with mitigated results However, it should be noted that up to 40% of women diagnosed with cervical cancer have been screened(18).

In this context, preventing cervical cancer through vaccination is particularly appealing. With a school-based program, we hope to reduce inequities and achieve a higher coverage than that achieved with screening(17,19). Only a few vaccination visits are required and interventions before pregnancy (and their potential impact) are prevented.

There are three HPV vaccines licensed in Canada. The first targets the 2 most oncogenic HPV types (bivalent, targeting types 16 and 18). The second targets these 2 viruses in addition to the 2 main types responsible of genital warts (quadrivalent, targeting types 6, 11, 16 and 18). The third licensed in February 2015 includes the 4 types in the 2nd vaccine in addition to 5 other oncogenic types (31, 33, 45, 52, and 58). The last two vaccines are produced by the same company (Merck) with the same manufacturing process. It is therefore reasonable to think that the nonavalent vaccine will likely replace the quadrivalent vaccine. Many prelicensure studies have compared the nonavalent to the quadrivalent vaccine. The results show that both vaccines generate a similar response against types 6,11,16,18. A 3-dose vaccine schedule is currently licensed for each vaccine (0, 2, 6 months for the nonavalent and quadrivalent vaccines and 0, 1, 6 months for the bivalent). For the 3 vaccines, a vaccine schedule with 2 doses administered 6 months apart is equally licensed in Canada in persons aged 9 to 14 years. These 3 vaccines are also licensed in many other countries, they are highly immunogenic and usually well tolerated(20–24). Randomized controlled trials have demonstrated their efficacy in the prevention of precancerous lesions(25,26). Maximal efficacy is achieved when vaccination is implemented before onset of sexual activity.

The vaccine used in this study is GARDASIL®, which was administered to the girls in the 4th year primary school (4th grade) targeted by this project.

1.3 HPV vaccination in Canada and in Quebec

All Canadian provinces have a school-based HPV vaccination program. In 2008, based on the recommendation of its provincial Advisory Board (Comité sur l'immunisation du Québec, CIQ) Quebec began a school-based vaccination program with Gardasil® and an extended HPV vaccination schedule in 4th grade (0, 6, 60 months) specifying that «the 3rd dose was to be administered … if deemed necessary». In 2012, most members of the HPV ad hoc committee (which included members of the Quebec Immunisation Advisory Board) renewed the recommendation on the use of the quadrivalent vaccine (Gardasil®). In spring 2013, the CIQ recommended not to administer the 3rd dose scheduled at month 60 and to evaluate (among others) the comparative efficacy and immunogenicity of two vaccine schedules (0, 6 months and 0, 6, 60 months) within a study (the present study)(27) .
Since 2016, in the province of Quebec, the nonavalent vaccine is used and the program offers HPV immunisation free of charge to grade 4 boys and to men who have sex with men (MSM) up to 26 years of age. (28,29).

In September 2018, the Quebec HPV vaccination public program implemented two main changes: 1) a mixed schedule is used in the program for boys and gilrs aged 9 to 17 years (one dose of nonavalent vaccine and one dose of vaccine bivalent) and 2) HPV vaccination is offered to boys in grade 9 (28).

1.4 HPV vaccination: benefit of alternative schedules

Alternative 2-dose vaccine schedules (e.g. 0, 6 months) offer obvious potential benefits: they can help to have a better acceptability while reducing vaccination-associated costs if further doses confer little or no additional benefit. Moreover, some vaccine schedules can facilitate coadministration with other vaccines. Based on current knowledge and data, it is relevant to consider 2-dose vaccine schedules.

HPV vaccines are very immunogenic and generate antibody titers higher than those conferred by the natural infection(30–32). Additionally, Canadian and Quebec data showed that children aged 9-13 years have a very good immune response. After 2 doses, they achieved antibody titers higher than that achieved in women aged 16-26 years who received 3 doses of vaccine and in whom the clinical efficacy of the vaccine has been demonstrated(33–35). Generally, the peak of antibody titers occurs 1 month following vaccination, then the titers decline during the next months and finally stabilize(36–39).

It might be useful to underscore that no antibody level has currently been identified as a reliable marker of vaccine efficacy. The duration of protection conferred by licensed vaccines is also unknown. Several cost-benefit studies have underscored that duration of protection is one of the key elements for the relevance of HPV vaccination programs(40). Therefore, the administration of a booster dose can be of interest. The administration of a booster dose 5 years after primary vaccination induces higher antibody titers than after primary vaccination. This has been seen with both hepatitis B and HPV vaccines(41,42). With this type of schedule (0, 6, 60 months), the high level of antibodies after the booster dose will occur shortly before the beginning of sexual activity in most youths. The booster will be administered at a time when youths can be reached easily (before they leave school) therefore maximizing the use of resources already in place for the vaccination program(13).

For these reasons, some health jurisdictions have adopted alternative vaccine schedules. In 2010, British Colombia (as well as Mexico) implemented a program similar to the Quebec program, offering the possibility to reconsider the need of a 3rd dose(43). In 2013, British Colombia chose to administer the 3rd dose 3 years after the first 2 doses which were administered 6 months apart (0, 6, 36 months). In 2014, they ceased to give the 3rd dose thus remaining with a 2-dose schedule. In 2017-2018, one Canadian province was using the 3-dose schedule recommended by the manufacturer (0, 2, 6 months) whereas the others adopted the 2-dose schedule (0, 6 months). As of 2012, the Swiss expert committee recommended the implementation of a 2-dose schedule with the 2 doses administered at a 6-month interval(44) and England did the same in 2014(45,46). In 2014, other committees and organizations recommended a schedule with 2 doses administered 6 months apart in preadolescents(47–49). In 2017, around 65% of the worldwide HPV vaccination programs were using a 2-dose schedule(50).

1.5 Why is it essential to conduct a study on HPV vaccine schedules in Quebec now?

To our knowledge, there are currently no data on the comparative efficacy (vaccinated versus unvaccinated) of the originally licensed schedules (0, 1, 6 or 0, 2, 6 months) when administered to preadolescents. There is also no efficacy data originating from studies which used the schedules with 2 doses administered 6 months apart or an extended schedule (0, 6, 60 months). The data from some ecological studies which compared women scheduled to received 3 doses but instead receive less (1 or 2 doses) were published or presented(51–55) and suggest inconsistent findings regarding the need of the 3rd dose. Some indicate a good protection following the first dose whereas others indicate that protection is inversely proportional to the number of doses received. Because the persons receiving a limited number of doses might have different characteristics and risks factors (in clinical studies non-compliant persons are generally at higher risk of the outcome of interest) interpretation of the results was difficult and this has been highlighted by the authors themselves or by letters to the editor(54).

The research team in charge of an Indian study reported results on comparative efficacy after 1, 2 or 3 doses of Gardasil administered to girls aged 9 to 18 years. The results of this study showed that geometric mean titers (GMT) at 7, 18, 24, 36 and 48 months in the group vaccinated with 2 doses were non-inferior to GMTs observed in the group receiving 3 doses. Antibody avidity at 18 months was similar in the groups vaccinated with 1, 2 and 3 doses. In their conclusion, the authors mentioned that the 2-dose schedule (0, 6 months) was non-inferior to the 3-dose schedule(56–58).

A study using population registries in Denmark evaluated the risk of anogenital warts among 550,690 girls eligible to vaccination between 2006 and 2012. The authors concluded that (1) with the original schedule (0, 2 and 6 months), 3 doses were necessary to achieve complete protection against anogenital warts and that (2) a vaccine schedule with 2 doses administered 6 months apart could be as efficient as the original 3-dose schedule. These results were observed in girls aged less than 16 years as well as in those aged 16 years or more.

Following HPV vaccination, the role of the immune memory in the protection against clinical diseases remains poorly studied(59,60) and we cannot directly extrapolate the immune system's response to a booster dose to the response following an infection, which is generally localised.

As mentioned previously, there are Canadian data on the comparative efficacy of the 0, 6 months and 0, 2, 6 months schedules. There is also a Quebec study which measured the immunogenicity of the 0, 6, 42 months schedule(61,62). To our knowledge, there is no study which will provide comparative data on the immunologic non-inferiority of the 3-dose 0, 6, 60 months schedule compared to the schedule with 2 doses administered 6 months apart. Instead of investigating the role of a booster dose we could theoretically just wait for the occurrence of lesions in vaccinated persons (breakthrough) and then confirm the need for a booster. However, that would entail a functional surveillance system with valid measurements occurring at different time points, which is not the situation in Quebec. Additionally, there can be a long delay from HPV infections to the lesions that can lead to cancer; therefore if we were to discover that duration of protection is shorter than expected it would be too late to intervene. Finally, even if the available tools (e.g. vaccination registry, screening and diagnostic and treatment information) made such a careful follow-up possible, in the scenario where lesions would occur in a higher number of vaccinees than expected, the decision-makers would not have the information on the relevance of an additional dose administered 5 years later nor would they know if this dose can significantly extend the duration of protection. In the absence of other data, they might have to come back to a schedule with 3 doses administered within 6 months.

1.6 Overlap between ICI-VPH and the Canadian study (QUEST) comparing a 2-dose schedule (0, 6 months) to a 3-dose schedule (0, 2, 6 months).

An ongoing Canadian study (QUEST) also aims to compare 2 vaccine schedules (see Appendix A). It is an observational quasi-experimental study which compares the cumulative risk of persistent HPV infections in young girls who received 2 (Quebec, British Colombia) or 3 doses of HPV vaccine (British Colombia, Alberta, Nova Scotia, Prince Edward Island) as part of their provincial school-based vaccination programs. This study does not provide an answer to the question regarding the vaccine schedule that can be used in Quebec: should we administer a 3rd dose at 60 months? Additionally, in the other provinces the vaccinated girls are older (11 – 13 years) than those vaccinated in Quebec (9 – 10 years). Given that only Quebec and British Colombia administered the first 2 doses at a 6-month interval during the recruitment period, Quebec's collaboration is essential to the success of the Canadian project. The data collected from the participants of the 2-dose group described in this protocol will be anonymously shared with the QUEST team. Of note, outcomes and effect measures have been harmonized between the 2 studies in order to facilitate collaboration. Part of the funding of the present study is provided by the Michael Smith Foundation, however most of the funding will be provided by the Quebec ministry of health (Ministère de la Santé et des Services sociaux du Québec, MSSS).

2. Objectives

2.1 Primary objective

To evaluate if a vaccine schedule with 2 doses of Gardasil® administered at a 6-month interval is non-inferior to a schedule with 3 doses administered at 0, 6, 60 months in the prevention of HPV-16 and HPV-18 infections which persist at least 6 months, and this, up to 10 years following the first injection.

2.2 Secondary objectives

a) To compare the antibody geometric mean titers and seropositivity (for HPV types 6, 11, 16, 18) among girls who received 2 doses of Gardasil® (0, 6 months) to the antibody geometric mean titers and seropositivity among girls who received 3 doses of Gardasil® (0, 6, 60 months) and this, 60, 90 and 120 months after the first injection.

b) To evaluate if a vaccine schedule with 2 doses of Gardasil® administered at a 6-month interval is non-inferior to a schedule with 3 doses administered at 0, 6, 60 months in the prevention of HPV-16 and HPV-18 infections which persist at least 6 months, and this, up to **13 years** following the first injection.

c) To compare the antibody geometric mean titers and seropositivity (for HPV types 6, 11, 16, 18, **31, 33, 45, 52 and 58**) among girls who received 2 doses of Gardasil® (0, 6 months) to the antibody geometric mean titers and seropositivity among girls who received 3 doses of Gardasil® (0, 6, 60 months) and this, 60, 90 and 120 months after the first injection.

d)To explore if a vaccine schedule with 2 doses of Gardasil® administered at a 6-month interval is non-inferior to a schedule with 3 doses administered at 0, 6, 60 months in the prevention of condylomas, and this, up to **13** years after the first injection..

e) To compare the level of persistent HPV infections in the 2 study groups to that observed in retrospective cohorts of unvaccinated girls.

3. Proposed study

3.1 Study design

We propose a non-inferiority randomized trial. The girls who received 2 doses of Gardasil® 5 years ago (see study population section) will be randomly assigned to a «2-dose» group where they will not receive additional doses of vaccines, or to a «3-dose» group where they will receive a 3rd dose of vaccine. The girls and their parents will know the group to which they were assigned. Blood samples will be collected among a subgroup of girls (250 in each of the two groups) in order to determine antibody titers (objectives 2.2.a and c). The persons assessing the primary and secondary outcomes (HPV and antibody testing) and the data analysts (statisticians) will be blinded to group allocation.

3.2 Study population

We will invite girls who initiated vaccination with Gardasil® in 2008, 2009, 2010 and 2011 at 9-10 years old (see explanation in section 38.4), who received 2 doses at a 6-month interval (minimum of 4 months and maximum of 12 months between doses) and are living in the Montreal, Quebec city (Capitale-Nationale) or Saguenay-Lac-Saint-Jean regions at the time of enrolment to participate to this study (the enrolment areas will be defined using the postal codes closest to the participating research centers). The girls will be invited about 5 years after the beginning of

vaccination, in such a way that during fall 2013 we will start inviting the girls who initiated vaccination in the fall 2008 as part of the school-based vaccination program. In 2014, we will invite the girls who initiated vaccination during fall 2009, in 2016 we will invite those who initiated vaccination during fall 2010, and in 2016 we will invite those who initiated vaccination during fall 2011 (see figure on conduct of the study, appendix B).

The only inclusion criteria are:

- To have received 2 doses of Gardasil® at a 6-month interval (minimum of 4 months and maximum of 12 months between doses) in 4th grade in 2008-2009, 2009-2010, 2010-2011 or 2011-2012;
- To understand French or English;
- To give an informed consent (girl and parent);
- To live in one of the targeted regions;
- To not be pregnant at the time of enrolment;
- To not be considered as immunodeficient at the time of enrolment, and at vaccination in 4th grade.

3.3 Enrolment

After approval by the Commission d'Accès à l'Information we will contact the Quebec health insurance board (Régie de l'Assurance Maladie du Québec, RAMQ) in order to obtain the contact information of girls aged 9 years on September 30th, 2008 (i.e. born between October 1st, 1998 and September 30th, 1999 for the first enrolment year), 9 years on September 30th, 2009 (i.e. born between October 1st, 1999 and September 30th, 2000 for the second enrolment year), 9 years on September 30th, 2010 (i.e. born between October 1st, 2000 and September 30th, 2001 for the third enrolment year), 9 years on September 30th, 2011 (i.e. born between October 1st, 2001 and September 30th, 2002 for the fourth enrolment year).

From September 2013 to June 2014 we will contact the girls' parents by mail to invite those who received 2 doses of Gardasil® during the 2008-2009 academic year. The letter will explain the purpose of the project, how it will be carried out and details on inclusion criteria. This same step will be carried out in 2014, 2015 and 2016.

A phone number and an e-mail address will enable parents to contact a research nurse (1) to obtain further information, (2) to check eligibility and (3) to take an appointment with a research nurse at the coordinating center.

During this appointment, the research nurse will confirm the participant's eligibility, will obtain informed consent from the girl and her parent, will perform a urine pregnancy test on the girl and will randomize her to one of the groups, administer the 3rd dose if she was assigned to the «3-dose» group and collect the blood sample if she is part of the subgroup targeted by the secondary objective on immunogenicity. A financial compensation will be offered to the girl and parent 20,00$ each) for this face-to-face appointment. The participant will collect the first HPV vaginal sample after receiving explanations on the self-sampling procedure and will fill the first questionnaire. A financial compensation will be provided for each self-sampling kit received (5,00$ per sampling, then 10,00$ per sampling when the girl turns 18 years of age).

3.4 Conduct of the study

After the first visit, the girls will have to perform vaginal self-samplings every 6 months and fill an online questionnaire once a year. They will receive an e-mail or a text message (SMS) 2 weeks before the target date. The self-sampling kits will be sent by mail. A reminder (e-mail or SMS) will be sent if the tube is not returned or the questionnaire not filled 2 weeks after the target date. A

second reminder (e-mail, SMS or phone call) will be made 2 weeks later (i.e. 1 month after the target date).

The 500 girls included in the immunogenicity aspect of the study will be seen at the coordinating center 90 and 120 months after the first injection, in order to collect blood samples which will be used to measure presence and titers of antibodies against HPV. Because there were less losses to follow-up than expected, and in order to reduce the costs as much as possible while being able to meet the secondary objective on immunogenicity, the blood sampling at month 90 will be performed in only 350 participants (175 per group). Section 3.8.2 specifies the number of participants required at the end of the study to reach the statistical power needed for this objective.

3.5 Study variables and effect measures

3.5.1 Intervention: definition and measurement

The main variable of interest is the number of Gardasil® doses received. It will be determined experimentally and will only have 2 possible values: 2 doses or 3 doses, the 3rd dose being the one administered by the research nurse during the face-to face-appointment. In order to have non-inferiority estimates as conservative as possible, a «per protocol» approach will be used during analyses as recommended for non-inferiority randomized trials(63). Participants who did not receive the number of doses planned in the protocol will be excluded from this analysis. This number will likely be limited given that the 3rd dose is given immediately after randomization. The experience with the school-based program show that very few girls randomized to the «2 doses» would have already received a 3rd dose (or more) out of the provincial public vaccination program (e.g. paying for an additional dose at a CLSC or a medical clinic during the 4 years following administration of the 2 first doses in 4th grade) (Manale Ouakki's personal communication, source: VAXIN database, Capitale-Nationale region). Girls will be regularly questioned on the subject and will be excluded from the per protocol analysis in case they have received an off-protocol dose of Gardasil®, Gardasil® 9 or Cervarix®. However, an intention-to-treat analysis will equally be performed and will include them (see section 3.9 analyses).

3.5.2 Primary outcome: definition and measurement

The primary outcome will be the cumulative incidence of persistent HPV 16 or HPV 18 infections. The ultimate goal of HPV vaccination is to reduce cervical cancer's incidence and mortality. However, these reductions are only expected in 30, maybe 40 years. It is not desirable to wait that long before evaluating and if necessary adjusting the vaccination program. Therefore persistent infection is the surrogate outcome preferred by many organisations (e.g. WHO) when decisions on efficacy have to be taken in a relatively short time (10 years)(64,65). A persistent infection predicts the risk of precancer very well and is relatively easy to measure and replicate.

A persistent infection will be defined as the presence of HPV 16 or HPV 18 DNA in 2 specimens of vaginal self-samples over 2 consecutive samplings. The samples will be collected every 6 months. Self-sampling is done with a swab inserted in the vagina then stored in an empty vial, before being sent through postal mail. This process has been successfully used by various teams in similarly aged populations(66).

In this definition of persistent infection, we will accept 2 consecutive samples performed at a minimum of 5-month interval and a maximum of 15-month interval. Two-step HPV DNA identification will be performed to minimize the costs. First, a generic test will be performed in order to check if HPV DNA is present. Then a Linear Array test will be performed on positive samples in order to identify the type(s) of HPV. Each type will be counted separately.

More precisely: a dry swab collected from each participant will be eluted in TE buffer. Obtained Cells are lysed (proteinase K 200ug, Tween 20 0,8%, incubated 90 min 65°C) and used as is for amplification. If lysed sample doesn't amplify, DNA is purified using the Master pure™ protocol (Epicentre, Madison, WI)(67–69). The DNA from the samples will first be submitted to an amplification protocol and generic detection using non-radioactive markers validated by our team(70,71). The DNA of the HPV present in the samples will be amplified by standardized biotinylated primers PGMY09/PGMY11 in a TC9700 thermocycler initially set a 95°C for 9 minutes, then at 95°C for 40 cycles of one minute, 1 minute at 55°C, and 1 minute at 72°C. Amplification will be completed by an extension step of 5 minutes at 72°C. The amplified products or amplicons will next be detected by a mix of digoxigenin-labeled probes, which will enable detection of genital genotypes without specifying the implicated type(s). The digoxigenin-labeled probe will be synthesized by amplification of certain HPV types with specific probes as previously described(71). Of note, ALL genital types can be detected with these probes, including those which are not used to synthesize the probes. The detection of the generic hybridized probes with the amplicons will be performed in 96 well microplates with standardized commercial reagents of the commercial kit PCR-ELISA DIG Detection kit (Roche Molecular Biochemicals, Indianapolis, IN) using the manufacturer's protocol and incorporating the modifications suggested and published by our team(70). These modifications include: hybridization of 20 ul of denatured amplicons with a generic probe in a 200ul hybridization solution for 3 hours at 42°C. A specimen will be considered positive if the absorption measured at A405 is ≥0.2 and negative if it is <0.2.

Specimens positive with the generic test will be submitted to a coamplification with ß-globin specific primers and PGMY09/PGMY11 standardized primers in a TC9700 thermocycler initially set a 95°C for 9 minutes, then at 95°C for 40 cycles of one minute, 1 minute at 55°C, and 1 minute at 72°C(72–74). Amplification will be completed by an extension step of 5 minutes at 72°C. Genotyping will be performed by a reverse hybridization reaction of the biotinylated amplicons generated by the prior reaction with probes fixed on a strip (called « array ») and revealed by a colorimetric reaction. We have successfully participated to an international quality control test organized by the World Health Organisation (WHO) on HPV genotyping. Genotyping of the 36 genotypes includes types: 6, 11, 16, 18, 26, 31, 33, 34, 35, 39, 40, 42, 44, 45, 51, 52 (cross-reacting probes), 53, 54, 56, 58, 59, 61, 62, 66, 67, 68, 69, 70, 71, 72, 73, 81, 82 (two variants including IS39), 83, 84 and 89. Samples positive with the probe used for detection of type 52 and also reacting with the probe of types 33, 35 or 58 will be analyzed with a specific probe for type 52 in real time PCR because linear array's HPV-52 probe also react with types 33, 35 and 58(75).

Samples negative for these genotypes and ß-globin positive will be considered HPV negative. Samples negative for HPV and ß-globin will be considered inadequate for PCR.

3.5.3 Secondary outcomes: definition and measurement

a) Immunogenicity:

A sub-sample of girls will be assigned to the part of the study measuring the immunogenicity and comparing seropositivity and antibody geometric mean titers (for HPV types 6, 11, 16, 18) in the two study groups.

The first 500 participants recruited will participate to the immunogenicity section of the study (250 per group). A blood sample will be collected at month 60, 90 and 120 following the beginning of vaccination in these participants. Five milliliters of blood will be collected in an 8.5 ml marbled-tube. Only 350 participants will have a blood sample at month 90.

A non-competitive serologic Luminex platform developed by the Quebec public health laboratory (Laboratoire de santé publique du Québec, LSPQ) will be used to detect and quantify HPV types' specific antibodies. This test is sensitive, replicable and quite simple to use(76,77). Depending of

the availability of the LSPQ Luminex platform, laboratory assays could be performed at the Centers for Disease Control and Prevention (CDC, Atlanta, USA) using multiplex direct IgG ELISA to HPV L1+L2 virus-like particles (VLPs) on Meso Scale Discovery platform as previously described with minor modification(78).

Clinical samples from the Vaccine Research Team CHU de Québec-Université Laval biobank (Nagano project #B12-06-986) will be used by the Frederick National Laboratory for Cancer Research (NIH/CDC) for the "9v HPV seronegatives to harmonize threshold" project to develop and validate HPV serological tests.

b) Primary outcome up to 13 years

HPV-16 and HPV-18 infections that persist at least 6 months will be measured, and this, up to **13 years** following the first injection as defined in 3.5.2.

c) Immunogenicity for 5 other types:

A sub-sample of girls will be assigned to the part of the study measuring the immunogenicity and comparing seropositivity and antibody geometric mean titers (for HPV types 6, 11, 16, 18 **and 31, 33, 45, 52, 58**) in the two study groups.

d) Condylomas:

Every year, the girls will be asked (via an online questionnaire) if a health professional told them they had condylomas. If the answer is yes, we will collect information (via a phone call by a research nurse) on the type and number of treatment(s) performed. Condylomas are not a reportable disease, it is therefore impossible to obtain information from registries. There is neither a specific claim code enabling us to collect this information via the Quebec health insurance database.

e) Comparison with retrospective cohorts of unvaccinated girls

Estimates of HPV 16 and HPV 18 persistent infections, will be obtained for various similar cohorts assembled before the beginning of vaccination in order to compared their rates to those obtained in ICI-VPH (see section on primary analysis). The same definition of persistent infection (as detailed in section 3.5.2) will be used.

3.5.4 Sample preservation and storage

Vaginal self-samples

The specimens obtained with the self-samplings will be kept at a 4°C temperature. The samples will then be frozen and kept in the laboratory for 15 years. They could be tested in other HPV studies. No test on the participants’ genetic profile will be performed using these samples.

Sera

The samples will be kept frozen at the research sites and during transportation to the Quebec public health laboratory (LSPQ) or the CDC laboratory for analyses.

The serum samples will be kept for 15 years. On the consent form, participants can agree to have their sample tested to identify antibodies against other vaccine preventable diseases. No genetic tests will be performed on these samples.

Study data will be kept for 25 years, after which they will be destroyed.

3.6 Randomization

Randomization will be performed via a web service. It will be stratified by centre (Montréal, Saguenay-Lac-St-Jean and Quebec City). Blocks varying in size between 4 and 8 participants will be used within each stratum.

3.7 Bias protection

We have chosen not to administer placebo to the girls randomized to the «2 dose» group for many reasons.

First, given that it is primarily an evaluation of the vaccination program, we want to perform an evaluation as close as possible to the real world environment. The girls will know if they received 2 or 3 doses whether or not the 3rd dose at 60 months is offered as part of the Quebec program. The only way through which knowledge of the number of doses received can influence the primary outcome is if it modifies the sexual behavior. Some people were worried about the fact that vaccination against STI could lead to more risky sexual behaviour, resulting from a false sense of security. Fortunately, the data available today do not support this hypothesis(79–81).

The main outcome is the detection of a virus in cervical secretions; it is therefore quite unlikely that the knowledge of the study group can influence that in anyway. The professionals in charge of the laboratory tests will not have access to the participants' randomization group. Similarly, the persons that will analyze potential screenings test will not know the group to which the participants were assigned.

Taking into consideration the related costs, risks, and inconvenience (discomfort, side effects associated to injecting a placebo) and its very low utility in improving the validity of the study results; it did not seem ethical or useful to us to include a placebo for the control group. The primary outcome will be measurement of persistent infections and completion of annual questionnaires enabling us to control for sexual behaviors in the analysis.

3.8 Sample size and feasibility of enrolment and follow-up.

3.8.1 Sample size needed to answer the primary research question.

The sample size was calculated to test the primary hypothesis of non-inferiority of a schedule with 2 doses administered 6 months apart, compared to a 3-dose schedule (0, 6, 60 months). We fixed the power at 90% as recommended for non-inferiority trials(63). The calculations were based on a bilateral log rank test with alpha set at 0.05 (corresponding to an alpha of 0.025 for a unilateral non-inferiority test) which is close to a test based on a 95% confidence interval derived from a Cox model. The non-inferiority margin will be fixed at 5%. Based on the data available in multicentric clinical trials measuring the efficacy of a schedule with 3 closely administered doses, the cumulative incidence of persistent infections was assumed to vary from 10% to 20%(23,82) during a 5-year period and the efficacy of the 2-dose schedule is expected to vary from 91 to 95%(21,30,83). The long term efficacy of the 3-dose schedule is unknown but was evaluated at 95%, which corresponds to an annual cumulative incidence of persistent infections of 0.5 to 1% among vaccinated girls (Table 1). The 2-dose schedule could therefore be declared non-inferior if we observed a cumulative incidence of persistent infections of 1 to 2%. Table 1 summarizes the various scenarios that were evaluated to calculate the sample size.

In other words, if we agree to lose a maximum of 5% of the vaccine efficacy (VE) with a 2-dose schedule, in order to conclude that it is non-inferior, we would agree to have a VE ≥ 90% for the 2-dose schedule. Therefore with a rate of 10%, 15% and 20% of persistent infections in the absence

of vaccination, we estimate to respectively observe a rate of 0.5%, 0.75% and 1% of persistent infections in the group vaccinated with 3 doses and a non-inferiority accepting a maximum of 5, 8 and 10 persistent infections per 1000 participant among the group vaccinated with 2 doses.

We had initially set the sample size at 2,167 per group. Given an expected frequency of 25% of losses to follow-up during the study, this number will be close to an effective sample size of 1625 per group (2 167*0.75). We believe that this sample size is close to that obtained with the most likely assumptions a 94-95% efficacy for the 2-dose schedule and a cumulative incidence of 10-15% of persistent infections (in the absence of vaccination) during the whole study. The results obtained will regularly be compared to the initial hypotheses. According to the conclusions of this comparison, the expected statistical power will be re-calculated. Adjustments on the number of participants to recruit or on the duration of follow up could be considered depending on the funding available.

Table 1 : Sample size needed in each group for a power of 90%

	Baseline rate pf persistent infections								
	10%			15%			20%		
Definition of non-inferiority	3-dose Eff	2-dose Eff	Size of the group	3-dose Eff	2-dose Eff	Size of the group	3-dose Eff	2-dose Eff	Size of the group
95% to 90%	95% (0.5%)	95% (0.5%)	1291	95% (0.75%)	95% (0.75%)	858	95% (1%)	95% (1%)	641
		94% (0.6%)	2156		94% (0.9%)	1431		94% (1.2%)	1070
		93% (0.7%)	4066		93% (1.05%)	2699		93% (1.4%)	2015
		92% (0.8%)	9649		92% (1.2%)	6403		92% (1.6%)	4780
		91% (0.9%)	40525		91% (1.35%)	26886		91% (1.8%)	20067

* Values in red represent the expected cumulative incidence of persistent infection in each scenario.

Revision of the sample size in January 2016:

We have reviewed the data used in our initial calculation and we have tried to refine and update them according to the most recent data in the literature, and what was observed in the present study after 2.5 years of activity. The modifications and precisions are on 2 aspects:

1. Estimation of the cumulative incidence of persistent HPV 16 and HPV 18 infections **per age range**, in order to more precisely evaluate the benefits and costs resulting from **following less participants for a longer period** (1 additional sample corresponding to 6 additional months of follow up, 2 additional samples corresponding to 1 year of additional follow up, etc.)
2. The proportion of losses to follow-up was reduced given the data observed in the study after following the 1st group of recruited participants for 2.5 years.
3. Proposal to adjust the budget according to the various scenarios, the various numbers of participants to be recruited and followed up and the various follow-up times.

When updating the literature search(84), we have refined our estimation of the cumulative incidence of persistent HPV 16 and HPV 18 infections per age range. Also, we have evaluated that it will be better to recruit less participants and follow them for a longer period while keeping the same study hypotheses and desired statistical power.

Table 2 presents the cumulative incidence of persistent infections that it will be possible to achieve if the participants are followed up for a longer period. It helps to calculate the number of participants needed to keep the same statistical power.

Using the values observed after 2.5 years of follow up, the percentage of losses to follow up was reviewed and set at 0% during the year of enrolment, 1%, 2%, 3% and 4% for the next 4 years respectively and 5% as of the 5th year of follow up (Table 2). We will no longer apply a global percentage of 25% of losses to follow up at the end of the study.

To meet the primary objective of the study while keeping the same hypotheses (i.e. a 95% efficacy in the 3-dose group and a 94% efficacy in the 2-dose group) the sample size needed has been calculated for follow-ups varying from 5 to 10 years corresponding to a cumulative incidence of persistent infections of 15% to 39%. When applying the percentage of losses to follow up accrued since the 1st year of participation, the number of girls to be recruited has been recalculated (Table 3).

In other words, when considering the cumulative incidence of persistent infections per age and the revised percentage of losses to follow up, for a 95% efficacy in the 3-dose group, a 94% efficacy in the 2-dose group and the enrolment of 2420 girls per group with a 4-year follow up, we will have 23 persistent infections, the same number of persistent infections that could be achieved by recruiting 1667 girls per group with a 5-year follow up or 1372 girls with a 6-year follow up.

We will therefore try to recruit 3334 (1667 X 2) participants and maintain a 5-year follow up for all of them. If this objective could not be achieved even with an additional year of enrolment, we will consider an additional follow-up of 6 months or 1 year depending on the number recruited.

Update, August 20, 2018: we recruited and randomised 3364 participants. Recruitment completed.

Table 2. Estimated cumulative incidence of HPV16/18 persistent infections according to the participant's age

Follow up (years)	Age (years)	Cumulative incidence of HPV16/18 persistent infections (%)
0	15	0,09
1	16	1,48
2	17	3,41
3	18	6,10
4	19	9,83
**5**	**20**	15,02
**5,5**	**20,5**	17,62
**6**	**21**	20,21
**6,5**	**21,5**	22,81
**7**	**22**	25,40
8	23	30,68
9	24	35,95
10	25	39,68

Table 3. Sample size needed per group for a statistical power of 90% and number of participants to be recruited depending of the duration of follow up (participant's age), cumulative incidence of persistent infections per age and losses to follow up per year of follow up.

Follow-up	Age	Cumulative incidence of HPV16/18 persistent infections (%)	Losses to follow-up (%)	Effective sample size (per group)	Number of participants to be recruited (per group)
0	15	0,09	0		
1	16	1,48	1		
2	17	3,41	2		
3	18	6,10	3		
**4**	**19**	**9,83**	**4**	**2186**	**2420**
**5**	**20**	**15,02**	**5**	**1431**	**1667**
**5,5**	**20,5**	**17,62**	**5**	**1220**	**1496**
**6**	**21**	**20,21**	**5**	**1063**	**1372**
**6,5**	**21,5**	**22,81**	**5**	**942**	**1280**
**7**	**22**	**25,40**	**5**	**846**	**1210**

8	23	30,68	5	700	1054
9	24	35,95	5	598	948
10	25	39,68	5	541	903

3.8.2 Sample size for the immunogenicity section

Various options for the calculation of the sample size needed for the immunogenicity section were analyzed. Various laboratory tests (cLia, total IgG, neutralizing antibodies, etc.) were performed during previous studies on the topic, resulting in an important heterogeneity in seropositivity levels and antibody titers reported after 2 or 3 doses of Gardasil®(32,34,35).

Taking into consideration that there is no « gold standard » for the laboratory tests measuring antibodies against HPV and that the sensitivity of the tests currently used varies significantly from one test to another, it was decided not to base the sample size calculation on seropositivity. We have assumed appropriate to base our sample size calculation on the ratio of geometric mean titers (GMT) expected in the 2 study groups (GMT2/GMT3).

The lower limit of the GMT ratio's confidence interval was set at 0.5, given that it is generally the case in this type of study(85,86). Because of potential clinical impacts, and future public health decisions, we want to have enough statistical power to detect a ratio ≥ 0.65 between the groups. To demonstrate a ratio of 0.65 between GMT2/GMT3 with a lower limit higher than 0.5, a statistical power of 90% and a significance level of 5%; the sample size needed is a total of 138 participants assessed per group at the end of the study (Table 2).

Taking into consideration a full respect of the study protocol (per-protocol) of 80 to 90% and a loss to follow up of about 5% per year, the enrolment of 500 participants (250 per group) was deemed necessary to be able to establish non-inferiority between the two study groups with a statistical power ≥90 % (α = 5 %). The enrolment of 500 participants at the beginning of the study (month 60 post primary immunization) will enable us to have at least 138 participants assessed at the end of the study.

Table 2. GMT2/GMT3 ratio: non-inferiority test with a lower limit set at 0.5

Lower limit	Ratio	N per group
0.5	0.55	1034
0.5	0.6	284
0.5	0.65	138
0.5	0.7	84

3.8.3 Statistical power for the objectives on the impact on condylomas

The very low expected frequency of condylomas in both study groups predicts a low statistical power; this is why these are exploratory objectives. Ecological studies suggest a very high efficacy on the reduction of condylomas(87–89). However, there are no data available to calculate a sample size in order to check if a 2-dose schedule is non-inferior to a 3-dose schedule in the prevention of condylomas. Given that a questionnaire already has to be administered in order to collect

information on confounding factors, adding a question on the diagnosis of condylomas will result in almost no additional costs or inconvenience. On the other hand, we believe that this procedure will bring useful information on HPV vaccine schedules' impact on condylomas in Quebec. An information that is otherwise very difficult to obtain given that there are no reliable diagnostic code(s) to check this issue using administrative databases. Moreover, given that HPV genotyping will enable identification of HPV types 6 and 11, it will be possible to link the presence of these genotypes in vaginal secretions to the clinical diagnosis of condylomas.

3.8.4 Feasibility of enrolment and follow-up.

Every year, about 8,000 girls are vaccinated with 2 doses of HPV vaccine in the two main health regions (Montreal and Capitale-Nationale [Quebec city region]). We plan to increase the radius of enrolment to neighboring towns and towns in the vicinity (using the area covered by the CLSC of residence and postal codes) of the two research centres (e.g. Laval, Montérégie, Chaudières-Appalaches, Saguenay-Lac-Saint-Jean). This will enable an increase of the number girls that could be invited to participate. Enrolment will be done over 4 years. By inviting all the girls in a cohort (e.g. the first cohort will be those vaccinated in 2008-2009) with a 10% participation rate (14 500*0,10= 1 450), we will be able to achieve our enrolment objective. According to the assumptions detailed in section 3.8.1, and in order to fulfill our objective, we will have to accrue between 22 and 52 persistent infections. We will have to follow-up each cohort for a minimum of 5 years in order to accrue this number of outcomes.
Many strategies implemented in these studies to maintain the children's participation to the study will be repeated here (e.g. identifying more than one postal address and more than one phone number, sending SMS yearly, etc.). We will equally ask the participants' authorization to obtain their contact information from the Quebec health Insurance Board in case we have difficulties getting in touch with them.

3.9 Analysis

Descriptive analyses will be used to describe the participants and compare the two study groups.
Specifically, the following analyses will be performed for each of the objectives mentioned in section 2.
For the primary objective:
A survival analysis (time to event analysis) will be performed in order to answer the main study questions. This approach will take into consideration the different duration of follow-up resulting from the fact that the date of entry in the cohort will vary between participants. Time 0 will be defined as the date of randomization. The time of event will be defined as the first occurrence of a persistent infection. Participants with no persistent infection will be censored (right-censored) at: (a) loss to follow-up, (b) death or (c) end of the study whichever comes first. The preliminary analyses will include the comparison of the Kaplan-Meyer curves using a non-parametric test (*log-rank test, Wilcoxon*) describing how the proportion of participants developing a persistent infection varies over time in each group. The main non-inferiority analysis will rely on the Hazard ratio's (HR) 95% confidence interval. Adjusted analyses will take into consideration the potential confounding factors collected with the annual questionnaire. If the HR's upper limit is not higher than 1.05 (representing a 5% difference of efficacy between the two schedules), we will conclude to a non-inferiority of the 2-dose calendar.

This analysis will then be repeated with the « intention-to-treat » method to evaluate the differences generated by a change in the exposure's definition. Sensitivity analyses will also be performed to evaluate the impact of modifying the outcome's definition. Indeed, it is likely that some girls will not exactly respect the vaginal self-samplings' schedule. We will vary the primary outcome's definition (e.g. consider as persistent infection only if the sample were consecutive and 6 months apart, or

consider as persistent infection a situation where the same type of virus was identified in two consecutive samples regardless of the number of months between the samples).

For the secondary objectives on immunogenicity (objectives 2.2.a and c), two analyses will be performed to measure non-inferiority at month 120:
1) An intention-to-treat analysis (*ITT analysis*) in which all the serologic results available will be included.
2) A per protocol (*according to the protocol, ATP analysis*) which will only include the participants who respected all the procedures planned in the protocol related to the immunogenicity analysis.
All the statistical tests will be performed with a type I error set at 5%. Percentages of seropositivity will be compared with a Chi square or Fisher test. GMTs will be compared with a *t*-test.
Among seropositive samples, GMTs will be calculated for each of the 3 time points planned in the protocol. GMTs obtained in the two groups at different study points will be compared using an analysis of variance (ANOVA) and a Duncan's multiple range test performed with SAS version 9.1.

For the other secondary objective on condylomas (2.2.d), we will mainly perform descriptive analyses as described at the beginning of this section.

An exact poisson regression is planned at the end of the study to compare the persistent infections observed among the vaccinated girls of the two study groups to that of an historical cohort of unvaccinated girls. Therefore, we will be able to calculate the number of persistent infections prevented by vaccination i.e. the difference between the expected and observed number of persistent infections (2.2.e).

4. Study management

4.1 TEAM

The expertise of the team members is diverse and ensures that all the skills necessary for the success of the study are brought together. C. Sauvageau and M-H. Mayrand will serve as principal investigators. They will act as site leads for Quebec City and Montreal, respectively. M-H. Mayrand has successfully coordinated and participated in large multicenter trials (92–94). Her expertise in RCT methodology, as well as her clinical experience with the target population, will contribute to the study's success. C. Sauvageau has previously conducted a randomized trial comparing 2-dose versus 3-dose schedules for the bivalent HPV vaccine (95) and has collaborated on several other studies on HPV vaccination (85,96,97). For one of these studies, recruitment was carried out using data from the RAMQ, after receiving approval from the CAI (97). V. Gilca has conducted studies with various vaccines, including clinical trials for DTaP-Polio-Hib, influenza, pneumococcus, meningococcus, and long-term cohort studies on hepatitis A, hepatitis B, and HPV vaccines. M. Ouakki has performed statistical analyses for several vaccination projects, including clinical trials for influenza vaccines (98), hepatitis B (99), as well as vaccine efficacy studies (100) and vaccine side effect surveillance (101). F. Coutlée oversees a research laboratory specializing in the molecular analysis of infections caused by human papillomaviruses. This laboratory supports numerous epidemiological studies on the role of these viruses in human pathology and their transmission. The lab has developed and validated detection and quantification tests for papillomaviruses in anogenital samples from women and men (75,102–104). Several clinical evaluations of commercial HPV detection and genotyping tests have been completed in recent years (72,92,105–107). The LSPQ team has developed and validated an HPV serological test at the LSPQ. Following the sudden death of Michel Couillard on April 20, 2013, Mr. Christian Therrien from the serology department at the LSPQ will collaborate on this project as Michel's replacement. The LSPQ will conduct serological tests for detecting HPV antibodies. Depending on the availability of the LSPQ platform, some serological tests might be performed at the CDC reference laboratory in Atlanta. Gitika Panicker and Elizabeth R. Unger have validated this multiplex platform and have conducted and collaborated on several studies on the impact of HPV vaccination (78,108–110). M.

Dionne and N. Boulianne are recognized experts in the field of immunization and have conducted and supervised numerous studies, including clinical trials and studies on vaccination coverage measurement.

4.2 Coordination

The coordination responsibilities will be divided between the CRCHU of Quebec and the CRCHUM. The study's principal research coordinators (nurses) will be based at the CRCHU of Quebec, where they are already working on longitudinal cohort studies. Vaginal samples will be sent to the CRCHUM (to Dr. Mayrand's facilities) for analysis in Dr. Coutlée's laboratory. Blood samples will be sent to the LSPQ or the CDC for analysis. Databases will be developed by the Applied Research Unit (URCA) of the CHU Sainte-Justine Research Center and deployed at both the CRCHUM and the CRCHU of Quebec. Nominal data from the Quebec City site will be stored on a secure server.

In addition to the study's principal coordinator, each center will have a coordinator responsible for the procedures specific to their site. The contact details of the participants will be kept at the research center where the initial visit was conducted. The self-sampling kits sent to participants will include a pre-paid return envelope addressed to the research center at the CRCHUM (Dr. Mayrand’s facilities) and financial compensation ($5.00 up to age 17 and $10.00 starting at age 18, per sample). These shipments will originate from the coordinating center in Quebec City. Periodically, the samples will be transferred to Dr. François Coutlée's virology laboratory at the CRCHUM for analysis. After recruitment is complete, participant follow-up will be managed by the coordinating center in Quebec. This includes follow-up reminders via email or phone and other email communications.

4.3 Calendar

Protocol Submission: REB CRCHUM and CRCHU of Quebec, January 2013
Final Protocol Approval: February-March 2013
Start of CAI and RAMQ Procedures: December 2012
Expected Decisions from CER, CAI, and RAMQ: April 2013
RAMQ Data Acquisition: August 2013

<u>Vaccinated girls who started vaccination in 2008</u>
Mailings: September 2013–April 2014Call returns: September 2013–June 2014
Recruitment/Randomization Visits: September 2013–June 2014
End of Follow-Up: 2021

<u>Vaccinated girls who started vaccination in 2009</u>
Mailings: May 2014-April 2015
Call returns: May 2014–March 2015
Recruitment/Randomization Visits: May 2014-June 2015
End of Follow-Up: 2021

<u>Vaccinated girls who started vaccination in 2010</u>
Mailings: May 2015-April 2016
Call returns: May 2015-March 2016
Recruitment/Randomization Visits: May 2015-June 2016
End of Follow-Up: 2021

<u>Vaccinated girls who started vaccination in 2011</u>
Mailings: May 2016-December 2016
Call returns: May 2016-December 2016
Recruitment/Randomization Visits: May 2016-December 2016

End of Follow-Up: 2021

An interim analysis is planned 5 years after the recruitment of the first participants (2018).

Study closure, analysis, presentations, report, and publications: 2022.

5. Ethics considerations

There are specific ethical challenges related to the enrolment of minors. However, the enrolment of minor is essential to meet our primary research question. Girls aged 18 years or more are (1) not targeted by the vaccination program(20); (2) they do not have the same response to the vaccine as younger girls(25,26) and (3) on average, they have a sexual experience that is very different from younger teenagers(92). Our team has vast experience in the enrolment and retention of this type of participant(91,93–95). We will first contact the girls' parents and they will decide to present the project to their child or not. Then a member of the team (nurse) will make sure to obtain both the parents' and the girls' consent. Randomization is explained by the state of «clinical equilibrium» on the primary research question. The risks associated with the intervention are well known by the girls and their parents given that they have already received two doses of Gardasil®. These are mainly local side effects as redness and pain at the injection site(20).

Given the immunogenicity and efficacy data which are available, the primary research hypothesis is that a schedule with 2 doses administered at a 6 month interval is non-inferior to a 3-dose schedule (0, 6, 60 months). If on the contrary, the non-inferiority of the 2-dose schedule could not be demonstrated, the girls in the 2-dose group will be offered a 3rd dose free of charge at the end of the study or at any other time fixed by the research team and the monitoring committee.

5.1 Confidentiality

All identifying data will be stored on a secure and dedicated server, protected by a password. These files will be kept separate from all results. During recruitment, participants will be assigned a study code, which will subsequently be used to identify all their samples and questionnaires. The key linking the study code to the participants will be kept separately on a dedicated server at the coordinating center. Only the study's coordinators will have access to it.

5.2 External committee

An external committee for study overview and data monitoring will be set up. It will consist of methodologists and content experts. The committee will meet once a year.

6. Expected impact

The information collected in this study will first enable us to make an informed decision (relying on efficacy data) on the usefulness of a 3rd dose of HPV vaccine.

This study will also help to have a better knowledge of the role of antibody titers in the acquisition of HPV persistent infections and might contribute to the establishment of a protective threshold. HPV vaccination has been highly effective(25). Therefore, occurrence of infections among vaccinees was not high enough to measure an antibody level that could be considered as protective against HPV-related clinical outcomes (condylomas, precancer or cancer). The analysis of immunogenicity and efficacy of HPV vaccination within the same study is essential. This approach will enable the measurement of the immune response (antibody testing) following vaccination and following natural infection. The antibody level maintained when no natural infection was detected will equally be informative. These various elements might help to determine the level of antibody needed to prevent a persistent infection.

Finally, we will better describe sexual behaviors, some lifestyles, cervical cancer screening and link this information with the occurrence of persistent infections. There is no doubt that this better portrait will help improve strategies for the prevention of cervical cancer.

7. Extension : phase 2

Depending on available funding, the research team will plan a 3-year extension (phase 2) of this research project resulting in a total of 8 years of follow-up. The same objectives will be maintained until the participants are aged 22-23 years and the following objective will be added:

To explore if a schedule with 2 doses administered at 0, 6 months is non-inferior to a schedule with 3 doses administered at 0, 6, 60 months for the prevention of precancerous and cancerous lesions identified during cervical cancer screening and this until 13 year following the beginning of vaccination.

Update August 2018:

An extension will be made, with secondary objectives added in section 2.2. Essentially, we will ask girls recruited in the first three years of the project to extend their participation until 2021, until the girls in the last recruited cohort have reached the five-year follow-up planned with the primary objective. This extension will therefore only apply to the first 3 cohorts of girls recruited in the study. Girls recruited in 2013-2014 will be invited to continue the study for an additional three years, those recruited in 2014-2015 for two additional years and those recruited in 2015-2016 for an additional year. The follow-up will be 10 to 13 years and will end for all in 2021.

.

References

1. de Villiers EM, Gunst K. Characterization of seven novel human papillomavirus types isolated from cutaneous tissue, but also present in mucosal lesions. J Gen Virol. août 2009;90(Pt 8):1999-2004.

2. de Villiers EM, Fauquet C, Broker TR, Bernard HU, zur Hausen H. Classification of papillomaviruses. Virology. 20 juin 2004;324(1):17‑27.

3. Bouvard V, Baan R, Straif K, Grosse Y, Secretan B, El Ghissassi F, et al. A review of human carcinogens--Part B: biological agents. Lancet Oncol. avr 2009;10(4):321‑2.

4. Munoz N, Bosch FX, Castellsague X, Diaz M, de Sanjose S, Hammouda D, et al. Against which human papillomavirus types shall we vaccinate and screen? The international perspective. Int J Cancer J Int Cancer. 20 août 2004;111(2):278‑85.

5. Syrjanen K, Hakama M, Saarikoski S, Vayrynen M, Yliskoski M, Syrjanen S, et al. Prevalence, incidence, and estimated life-time risk of cervical human papillomavirus infections in a nonselected Finnish female population. Sex Transm Dis. janv 1990;17(1):15‑9.

6. Ho GY, Bierman R, Beardsley L, Chang CJ, Burk RD. Natural history of cervicovaginal papillomavirus infection in young women. N Engl J Med. 12 févr 1998;338(7):423‑8.

7. Moscicki AB, Schiffman M, Kjaer S, Villa LL. Chapter 5: Updating the natural history of HPV and anogenital cancer. Vaccine. 21 août 2006;24 Suppl 3:S42-51.

8. Hildesheim A, Schiffman MH, Gravitt PE, Glass AG, Greer CE, Zhang T, et al. Persistence of type-specific human papillomavirus infection among cytologically normal women. J Infect Dis. févr 1994;169(2):235‑40.

9. Koutsky LA, Holmes KK, Critchlow CW, Stevens CE, Paavonen J, Beckmann AM, et al. A cohort study of the risk of cervical intraepithelial neoplasia grade 2 or 3 in relation to papillomavirus infection. N Engl J Med. 29 oct 1992;327(18):1272‑8.

10. Ho GY, Burk RD, Klein S, Kadish AS, Chang CJ, Palan P, et al. Persistent genital human papillomavirus infection as a risk factor for persistent cervical dysplasia. J Natl Cancer Inst. 20 sept 1995;87(18):1365‑71.

11. Kjaer SK, van den Brule AJ, Paull G, Svare EI, Sherman ME, Thomsen BL, et al. Type specific persistence of high risk human papillomavirus (HPV) as indicator of high grade cervical squamous intraepithelial lesions in young women: population based prospective follow up study. BMJ. 14 sept 2002;325(7364):572.

12. Ouhoummane N, Goggin P, Louchini R. Les infections au virus du papillome humain (VPH) et le portrait des cancers associés à ces infections au Québec. Québec : Institut national de santé publique du Québec; 2013 p. 86 p. + annexes. Rapport no 978-2-550-68836-5.

13. Comité sur l'immunisation du Québec, Comité scientifique ad hoc VPH. La vaccination contre les VPH au Québec : mise à jour des connaissances et propositions du comité d'experts. Québec : Institut national de santé publique du Québec; 2012 p. 148 p.

14. Groupe de travail sur les lignes directrices pour le dépistage du cancer du col utérin au Québec. Lignes directrices sur le dépistage du cancer du col utérin au Québec. Québec : Institut national de santé publique du Québec; 2011 p. 40 p.

15. Jakobsson M, Gissler M, Paavonen J, Tapper AM. Loop electrosurgical excision procedure and the risk for preterm birth. Obstet Gynecol. sept 2009;114(3):504‑10.

16. Arbyn M, Kyrgiou M, Simoens C, Raifu AO, Koliopoulos G, Martin-Hirsch P, et al. Perinatal mortality and other severe adverse pregnancy outcomes associated with treatment of cervical intraepithelial neoplasia: meta-analysis. BMJ. 2008;337:a1284.

17. Goggin P, Mayrand MH. Avis sur l'optimisation du dépistage du cancer du col utérin au Québec. Québec : Institut national de santé publique du Québec; 2009 p. 101 p.

18. Spence AR, Goggin P, Franco EL. Process of care failures in invasive cervical cancer: Systematic review and meta-analysis. Prev Med. 22 juin 2007;45(2‑3):96‑106.

19. Ministère de la Santé et des Services sociaux. Campagne de vaccination en milieu scolaire contre le VPH. Flash Vigie. sept 2012;7(7):3‑4.

20. Ministère de la Santé et des Services sociaux. Protocole d'immunisation du Québec: ministère de la Santé et des Services sociaux. 2012. 447 p. p.

21. The Future II Study Group. Quadrivalent vaccine against human papillomavirus to prevent high-grade cervical lesions. N Engl J Med. 10 mai 2007;356(19):1915‑27.

22. Garland SM, Hernandez-Avila M, Wheeler CM, Perez G, Harper DM, Leodolter S, et al. Quadrivalent vaccine against human papillomavirus to prevent anogenital diseases. N Engl J Med. 10 mai 2007;356(19):1928‑43.

23. Paavonen J, Naud P, Salmeron J, Wheeler CM, Chow SN, Apter D, et al. Efficacy of human papillomavirus (HPV)-16/18 AS04-adjuvanted vaccine against cervical infection and precancer caused by oncogenic HPV types (PATRICIA): final analysis of a double-blind, randomised study in young women. Lancet. 25 juill 2009;374(9686):301‑14.

24. Stanley M. Prospects for new human papillomavirus vaccines. Curr Opin Infect Dis. févr 2010;23(1):70‑5.

25. Merck Frosst Canada ltée. GARDASIL - Vaccin recombinant quadrivalent contre le virus du papillome humain (types 6, 11, 16 et 18). Québec : Merck Frosst Canada Ltée; ao 2011 p. 63 p.

26. GlaxoSmithKline. CERVARIX - Human Papillomavirus vaccine Types 16 and 18 (Recombinant, AS04 adjuvanted). Québec : GlaxoSmithKline; ao 2011 p. 52 p.

27. Sauvageau C, Gilca V. La vaccination des préadolescents contre les virus du papillome humain (VPH) au Québec : deux ou trois doses? Québec : Institut national de santé publique du Québec; 2013 p. 60 p. + annexes. Rapport no ISBN : 978-2-550-68492-3.

28. [En ligne]. Ministère de la Santé et des Services sociaux. Protocole d'immunisation du Québec. 7e édition, [En ligne]. http://www.msss.gouv.qc.ca/professionnels/vaccination/protocole-d-immunisation-du-quebec-piq/.

29. [En ligne]. Ministère de la Santé et des Services sociaux. Modification au Programme de vaccination contre les virus du papillome humain - Ajout de la vaccination gratuite contre les virus du papillome humain des hommes âgés de 26 ans et moins ayant des relations sexuelles avec d'autres hommes et de la vaccination des garçons de la 4e année du primaire [En ligne] : http://www.msss.gouv.qc.ca/professionnels/vaccination/documents/piq/nouveautes-decembre-2015.pdf (Page consultée le 19 janvier 2016).

30. Villa LL, Costa RL, Petta CA, Andrade RP, Ault KA, Giuliano AR, et al. Prophylactic quadrivalent human papillomavirus (types 6, 11, 16, and 18) L1 virus-like particle vaccine in young women: a randomised double-blind placebo-controlled multicentre phase II efficacy trial. Lancet Oncol. mai 2005;6(5):271‑8.

31. Harper DM, Franco EL, Wheeler C, Moscicki AB, Romanowski B, Roteli-Martins CM, et al. Sustained efficacy up to 4.5 years of a bivalent L1 virus-like particle against human papillomavirus types 16 and 18: follow-up from a randomised control trial. Lancet. 6 avr 2006;367(9518):1247‑55.

32. Dobson S, Dawar M, Scheifele D, Kollmann T, McNeil S, Halperin S, et al. Are two doses of HPV vaccine adequate in girls? Oral presentation. 25th International Papillomavirus Conference. Malmo, Sweden, May 8-14, 2009.

33. Block SL, Nolan T, Sattler C, Barr E, Giacoletti KE, Marchant CD, et al. Comparison of the immunogenicity and reactogenicity of a prophylactic quadrivalent human papillomavirus (types 6, 11, 16, and 18) L1 virus-like particle vaccine in male and female adolescents and young adult women. Pediatrics. nov 2006;118(5):2135‑45.

34. Einstein MH, Baron M, Levin MJ, Chatterjee A, Edwards RP, Zepp F, et al. Comparison of the immunogenicity and safety of Cervarix and Gardasil human papillomavirus (HPV) cervical cancer vaccines in healthy women aged 18-45 years. Hum Vaccin. oct 2009;5(10):705‑19.

35. Dobson S, Dawar M, Money D, Bettinger J, Krajden M, Langley J, et al. Two dose vaccine trial of Q-HPV: results at 36 months. Oral presention. 27th International Papillomavirus Conference and Clinical Workshop, Berlin, Germany, September 17-22, 2011.

36. De Carvalho N, Teixeira J, Roteli-Martins CM, Naud P, De Borba P, Zahaf T, et al. Sustained efficacy and immunogenicity of the HPV-16/18 AS04-adjuvanted vaccine up to 7.3 years in young adult women. Vaccine. 31 août 2010;28(38):6247‑55.

37. Joura EA, Kjaer SK, Wheeler CM, Sigurdsson K, Iversen OE, Hernandez-Avila M, et al. HPV antibody levels and clinical efficacy following administration of a prophylactic quadrivalent HPV vaccine. Vaccine. 9 déc 2008;26(52):6844‑51.

38. Frazer IH. Measuring serum antibody to human papillomavirus following infection or vaccination. Gynecol Oncol. juin 2010;118(1 Suppl):S8-11.

39. Olsson SE, Villa LL, Costa RL, Petta CA, Andrade RP, Malm C, et al. Induction of immune memory following administration of a prophylactic quadrivalent human papillomavirus (HPV) types 6/11/16/18 L1 virus-like particle (VLP) vaccine. Vaccine. 21 juin 2007;25(26):4931‑9.

40. Van de Velde N, Boily MC, Drolet M, Franco EL, Mayrand M-H, Kliewer EV, et al. Population-level impact of the bivalent, quadrivalent and nonavalent human papillomavirus vaccines: A model-based analysis. J Natl Cancer Inst. 104(22):1712‑23.

41. Duval B, Gilca V, Boulianne N, De Wals P, Masse R, Trudeau G, et al. Comparative long term immunogenicity of two recombinant hepatitis B vaccines and the effect of a booster dose given after five years in a low endemicity country. Pediatr Infect J. mars 2005;24(3):213‑8.

42. Duval B, Gilca V, Boulianne N, De Wals P, Trudeau G, Massé R, et al. HBs Antibody kinetics five years after booster vaccination with Engerix B. Poster presentation. 47th Interscience Conference on Antimicrobial Agents & Chemotherapy Medical Conference (ICAAC). Mc Cormick Place, Chicago, Illinois, September 17-20, 2007.

43. [En ligne]. Health Care Professional. Human Papillomavirus (HPV) Vaccine Extended Schedule for Girls between the ages of 9-13 years [On line] http://immunizebc.ca/sites/default/files/docs/HPVprofessionalQA_Jan2011_final.pdf (accessed January 3, 2013).

44. Commission fédérale pour les vaccinations (CFV), Office fédéral de la santé publique (OFSP). Vaccination contre les VPH: passage du shéma à trois doses au schéma à deux doses chez les adolescentes âgées de moins de 15 ans. Mal Transm. 6 févr 2012;Bulletin 6:106‑10.

45. [En ligne]. Committee on Vaccination and Immunisation. Minute of the meeting on Tuesday 11 and Wednesday 12 February 2014 [On line] https://www.gov.uk/government/groups/joint-committee-on-vaccination-and-immunisation (Page accessed June 16, 2014).

46. [En ligne]. Public Health England. HPV vaccination programme: change from 3 to 2 doses [On line] https://www.gov.uk/government/publications/schedule-change-from-3-to-2-doses-in-the-hpv-vaccination-programme (Page accessed June 16, 2014).

47. European Medicine Agency. Gardasil human papillomavirus vaccine [types 6, 11, 16, 18] (recombinant, adsorbed). London : European Medicine Agency; 2014 p. 1‑4.

48. [En ligne]. PR Newswire. Gardasil®: New 2-dose Schedule Granted Positive CHMP Opinion for Europe’s Leading HPV Vaccine [On line] http://www.prnewswire.co.uk/news-releases/gardasil-new-2-dose-schedule-granted-positive-chmp-opinion-for-europes-leading-hpv-vaccine-247445321.html (Page accessed April 30, 2014).

49. World Health Organisation. Meeting of the Strategic Advisory Group of Experts on immunization, April 2014 -- conclusions and recommendations. Wkly Epidemiol Rec. 2014;89(21):221‑36.

50. Brotherton JML, Bloem PN. Population-based HPV vaccination programmes are safe and effective: 2017 update and the impetus for achieving better global coverage. Best Pract Res Clin Obstet Gynaecol. 6 sept 2017;47:42‑58.

51. Mahmud SM, Kliewer EV, Demers AA, Lambert P, Templeton K, Harrison M, et al. Quadrivalent HPV vaccination and the incidence of cervical dysplasia in Manitoba, Canada. Oral presentation. 28th International Papillomavirus Conference, San Juan, Puerto Rico, November 30 - December 6, 2012.

52. Kruger Kjaer S, Blomberg M, Munk C, Dehlendorff C. Strongly decreased risk of genital warts after vaccination against HPV – Nationwide followup of vaccinated and non-vaccinated women in Denmark. Oral presentation. 28th International Papillomavirus Conference, San Juan, Puerto Rico, November 30 - December 6, 2012.

53. Crowe E, Pandeya N, Brotherton JM, Dobson AJ, Kisely S, Lambert SB, et al. Effectiveness of quadrivalent human papillomavirus vaccine for the prevention of cervical abnormalities: case-control study nested within a population based screening programme in Australia. BMJ. 2014;348:g1458.

54. Markowitz LE, Drolet M, Perez N, Jit M, Brisson M. Human papillomavirus vaccine effectiveness by number of doses: Systematic review of data from national immunization programs. Vaccine. 06 2018;36(32 Pt A):4806‑15.

55. Markowitz L. High effectiveness after vaccine type prevalence after 1, 2, and 3 doses of quadrivalent HPV vaccine, United States, HPV 2017, 2 March 2017.

56. Sankaranarayanan R, Prabhu PR, Pawlita M, Pillai MR, Bhatla N. Comparison of immunogenicity following a single, two or three doses of human papilloma virus vaccination in India. 30th International Papillomavirus Conference & Clinical and Public Health Workshops (oral presentation), Lisbon, Portugal, September 17-21, 2015.

57. Sankaranarayanan R, Prabhu PR, Pawlita M, Gheit T, Bhatla N, Muwonge R, et al. Immunogenicity and HPV infection after one, two, and three doses of quadrivalent HPV vaccine in girls in India: a multicentre prospective cohort study. Lancet Oncol. janv 2016;17(1):67‑77.

58. Sankaranarayanan R, Joshi S, Muwonge R, Esmy PO, Basu P, Prabhu P, et al. Can a single dose of human papillomavirus (HPV) vaccine prevent cervical cancer? Early findings from an Indian study. Vaccine. 06 2018;36(32 Pt A):4783‑91.

59. Stanley M. Potential mechanisms for HPV vaccine-induced long-term protection. Gynecol Oncol. juin 2010;118(1 Suppl):S2-7.

60. Stanley M. Introduction. The human papillomavirus VLP vaccines. Gynecol Oncol. juin 2010;118(1 Suppl):S1.

61. Gilca V, Sauvageau C, Boulianne N, De Serres G, Couillard M, Krajden M, et al. Immunogenicity of quadrivalent HPV and combined hepatitis A and B vaccine when co-administered or administered one month apart to 9-10 year-old girls according to 0-6 month schedule. Hum Vaccines Immunother. 2014;10(8):2438‑45.

62. Gilca V, Sauvageau C, Boulianne N, De Serres G, Crajden M, Ouakki M, et al. The effect of a booster dose of quadrivalent or bivalent HPV vaccine when administered to girls

previously vaccinated with two doses of quadrivalent HPV vaccine. Hum Vaccin Immunother. 2015;11(3):732‑8.

63. Piaggio G, Elbourne DR, Altman DG, Pocock SJ, Evans SJ. Reporting of noninferiority and equivalence randomized trials: an extension of the CONSORT statement. JAMA. 8 mars 2006;295(10):1152‑60.

64. Pagliusi SR, Teresa Aguado M. Efficacy and other milestones for human papillomavirus vaccine introduction. Vaccine. 16 déc 2004;23(5):569‑78.

65. Wacholder S. Chapter 18: Statistical issues in the design and analysis of studies of human papillomavirus and cervical neoplasia. J Natl Cancer Inst Monogr. 2003;(31):125‑30.

66. Quincy BL, Turbow DJ, Dabinett LN. Acceptability of self-collected human papillomavirus specimens as a primary screen for cervical cancer. J Obstet Gynaecol. janv 2012;32(1):87‑91.

67. Tran-Thanh D, Provencher D, Koushik A, Duarte-Franco E, Kessous A, Drouin P, et al. Herpes simplex virus type II is not a cofactor to human papillomavirus in cancer of the uterine cervix. Am J Obstet Gynecol. janv 2003;188(1):129‑34.

68. Koushik A, Ghosh A, Duarte-Franco E, Forest P, Voyer H, Matlashewski G, et al. The p53 codon 72 polymorphism and risk of high-grade cervical intraepithelial neoplasia. Cancer Detect Prev. 2005;29(4):307‑16.

69. Hamlin-Douglas LK, Coutlee F, Roger M, Franco EL, Brassard P. Prevalence and age distribution of human papillomavirus infection in a population of Inuit women in Nunavik, Quebec. Cancer Epidemiol Biomark Prev. nov 2008;17(11):3141‑9.

70. Legault V, Burchell A, Goggin P, Nicolau B, Brassard P, Guenoun J, et al. Generic microtiter plate assay for triaging clinical specimens prior to genotyping of human papillomavirus DNA via consensus PCR. J Clin Microbiol. nov 2011;49(11):3977‑9.

71. Kornegay JR, Shepard AP, Hankins C, Franco E, Lapointe N, Richardson H, et al. Nonisotopic detection of human papillomavirus DNA in clinical specimens using a consensus PCR and a generic probe mix in an enzyme-linked immunosorbent assay format. J Clin Microbiol. oct 2001;39(10):3530‑6.

72. Coutlee F, Rouleau D, Petignat P, Ghattas G, Kornegay JR, Schlag P, et al. Enhanced detection and typing of human papillomavirus (HPV) DNA in anogenital samples with PGMY primers and the Linear array HPV genotyping test. J Clin Microbiol. juin 2006;44(6):1998‑2006.

73. de Pokomandy A, Rouleau D, Ghattas G, Vezina S, Cote P, Macleod J, et al. Prevalence, clearance, and incidence of anal human papillomavirus infection in HIV-infected men: the HIPVIRG cohort study. J Infect Dis. 1 avr 2009;199(7):965‑73.

74. Petignat P, Hankins C, Walmsley S, Money D, Provencher D, Pourreaux K, et al. Self-sampling is associated with increased detection of human papillomavirus DNA in the genital tract of HIV-seropositive women. Clin Infect Dis. 15 août 2005;41(4):527‑34.

75. Coutlee F, Rouleau D, Ghattas G, Hankins C, Vezina S, Cote P, et al. Confirmatory real-time PCR assay for human papillomavirus (HPV) type 52 infection in anogenital specimens screened for HPV infection with the linear array HPV genotyping test. J Clin Microbiol. nov 2007;45(11):3821‑3.

76. Opalka D, Matys K, Bojczuk P, Green T, Gesser R, Saah A, et al. Multiplexed serologic assay for nine anogenital human papillomavirus types. Clin Vaccine Immunol. mai 2010;17(5):818‑27.

77. Gilca V, Sauvageau C, Krajden M, Cook D, Dagenais C, Therrien C, et al. Comparison of different immunoassays used for HPV antibody assessment in vaccinated and unvaccinated individuals. Poster presentation. 29th Annual International Papillomavirus Conference and Public Health & Clinical Workshops, Seattle, USA, 20-25 August, 2014.

78. Panicker G, Rajbhandari I, Gurbaxani BM, Querec TD, Unger ER. Development and evaluation of multiplexed immunoassay for detection of antibodies to HPV vaccine types. J Immunol Methods. févr 2015;417:107‑14.

79. Bednarczyk RA, Davis R, Ault K, Orenstein W, Omer SB. Sexual Activity-Related Outcomes After Human Papillomavirus Vaccination of 11- to 12-Year-Olds. Pediatrics. 15 oct 2012;130(5):798‑805.

80. Forster AS, Marlow LA, Stephenson J, Wardle J, Waller J. Human papillomavirus vaccination and sexual behaviour: cross-sectional and longitudinal surveys conducted in England. Vaccine. 13 juill 2012;30(33):4939‑44.

81. Mather T, McCaffery K, Juraskova I. Does HPV vaccination affect women's attitudes to cervical cancer screening and safe sexual behaviour? Vaccine. 2 mai 2012;30(21):3196‑201.

82. Steben M for the Quadrivalent HPV Vaccine Efficacy Trial Team,. Analysis of quadrivalent HPV vaccine efficacy against HPV 16/18 persistent infection in both men and women. Abstract presented at Eurogin 2011 Congress, Lisbon, May 8-11, 2011.

83. Medeiros LR, Rosa DD, da Rosa MI, Bozzetti MC, Zanini RR. Efficacy of human papillomavirus vaccines: a systematic quantitative review. Int J Gynecol Cancer. oct 2009;19(7):1166‑76.

84. Smith MA, Tellier PP, Roger M, Coutlee F, Franco EL, Richardson H. Determinants of human papillomavirus coinfections among Montreal university students: the influence of behavioral and biologic factors. Cancer Epidemiol Biomark Prev. mai 2014;23(5):812‑22.

85. Dobson S, Krajden M, Marra F, Miller D, Money D, Naus M, et al. A Controlled Trial to Assess the Immunogenicity of a Proposed Paediatric Dosing Schedule of Human Papillomavirus Vaccine. Vancouver : Government of BC; 2008 p. 46 p.

86. Ogilvie G, Dobson S, Krajden M, Money D, Dawar M, Naus M, et al. QUEST: Quadrivalent HPV Vaccine Evaluation Study. Vancouver : BC Centre for Disease Control; 2012 p. 27 p.

87. Fairley CK, Hocking JS, Gurrin LC, Chen MY, Donovan B, Bradshaw CS. Rapid decline in presentations of genital warts after the implementation of a national quadrivalent human

papillomavirus vaccination programme for young women. Sex Transm Infect. déc 2009;85(7):499‑502.

88. Donovan B, Franklin N, Guy R, Grulich AE, Regan DG, Ali H, et al. Quadrivalent human papillomavirus vaccination and trends in genital warts in Australia: analysis of national sentinel surveillance data. Lancet Infect Dis. janv 2011;11(1):39‑44.

89. Read TR, Hocking JS, Chen MY, Donovan B, Bradshaw CS, Fairley CK. The near disappearance of genital warts in young women 4 years after commencing a national human papillomavirus (HPV) vaccination programme. Sex Transm Infect. déc 2011;87(7):544‑7.

90. Gilca V, De Serres G, Boulianne N, De Wals P, Trudeau G, Masse R, et al. Antibody kinetics among 8-10 years old respondents to hepatitis B vaccination in a low endemic country and the effect of a booster dose given five or ten years later. Vaccine. 9 oct 2009;27(43):6048‑53.

91. Gilca V, De Serres G, Boulianne N, De Wals P, Murphy D, Trudeau G, et al. Antibody and immune memory persistence after vaccination of preadolescents with low doses of recombinant hepatitis B vaccine. Hum Vaccin. 11 févr 2010;6(2):212‑8.

92. Pica LA, Traoré I, Bernèche F, Laprise P, Cazale L, Camirand H, et al. L'Enquête québécoise sur la santé des jeunes du secondaire 2010-2011. Le visage des jeunes d'aujourd'hui : leur santé physique et leurs habitudes de vie, Tome 1. Québec : Institut de la statistique du Québec; 2012 p. 258 p.

93. Genevrois S, Godeaux O, Schuind A, Descamps D. Evaluation of the safety and immunogenicity of GlaxoSmithKline Biologicals' HPV vaccine 580299 when administered in healthy females aged 9-25 years using and alternative schedule and an alternative dosing as compared to the standard schedule and dosing. Belgium : GlaxoSmithKline; 2012 p. 120 p.

94. Gilca V, Sauvageau C, Dionne M, Boulianne N, De Serres G. Co-administration of Gardasil and Twinrix to 9-10 year-old girls preliminary immunogenicity data. Poster presentation. 26th International Papillomavirus Conference and Clinical and Public Health Workshops, Montréal, July 3-8, 2010. 2010.

95. Mayrand MH, Duarte-Franco E, Rodrigues I, Walter SD, Hanley J, Ferenczy A, et al. Human papillomavirus DNA versus Papanicolaou screening tests for cervical cancer. N Engl J Med. 18 oct 2007;357(16):1579‑88.

96. Goss PE, Ingle JN, Ales-Martinez JE, Cheung AM, Chlebowski RT, Wactawski-Wende J, et al. Exemestane for breast-cancer prevention in postmenopausal women. N Engl J Med. 23 juin 2011;364(25):2381‑91.

97. Abrahamowicz M, Bentley J, Coutlée F, Trottier H for the CoHIPP study team. Colposcopy vs. HPV testing to identify persistent precancers post treatment: The CoHIPP trial. American Society for Colposcopy and Cervical Pathology 2012 Biennial Meeting, San Fransciso, USA, March 2012.

98. Kiely M, Sauvageau C, Dubé E, Deceuninck G, De Wals P. Virus du papillome humain : connaissances, croyances et comportements des femmes québécoises. Can J Public Health. juill 2011;102(4):303-07.

99. Gilca V, De Serres G, Hamelin ME, Boivin G, Ouakki M, Boulianne N, et al. Antibody persistence and response to 2010-11 trivalent influenza vaccine one year after a single dose of 2009 AS03-adjuvanted pandemic H1N1 vaccine in children. 29th Annual Meeting of the European Society for Paediatric Infectious Diseases (ESPID), The Hague, The Netherlands, June 7-11, 2011.

100. Gilca V, De Serres G, Boulianne N, Murphy D, De Wals P, Ouakki M, et al. Antibody persistence and the effect of a booster dose given 5, 10 or 15years after vaccinating preadolescents with a recombinant hepatitis B vaccine. Vaccine. 7 janv 2013;31(3):448‑51.

101. De Wals P, Carbon M, Sévin É, Deceuninck G, Ouakki M. Reduced physician claims for otitis media after implementation of pneumococcal conjugate vaccine program in the province of Quebec, Canada. Poster presentation. 27th Annual Meeting of the European Society for Paediatric Infectious Diseases. Belgium, Brussels, June 9-13. 2009.

102. De Serres G, Gariepy MC, Coleman B, Rouleau I, McNeil S, Benoit M, et al. Short and Long-Term Safety of the 2009 AS03-Adjuvanted Pandemic Vaccine. PloS One. 2012;7(7):e38563.

103. Aho J, Hankins C, Tremblay C, Forest P, Pourreaux K, Rouah F, et al. Genomic polymorphism of human papillomavirus type 52 predisposes toward persistent infection in sexually active women. J Infect Dis. 1 juill 2004;190(1):46‑52.

104. Alvarez J, de Pokomandy A, Rouleau D, Ghattas G, Vézina S, Coté P, et al. Episomal and integrated human papillomavirus type 16 loads and anal intraepithelial neoplasia in HIV-seropositive men. AIDS Lond Engl. 24 sept 2010;24(15):2355‑63.

105. Azizi N, Brazete J, Hankins C, Money D, Fontaine J, Koushik A, et al. Influence of human papillomavirus type 16 (HPV-16) E2 polymorphism on quantification of HPV-16 episomal and integrated DNA in cervicovaginal lavages from women with cervical intraepithelial neoplasia. J Gen Virol. juill 2008;89(Pt 7):1716‑28.

106. Dufresne S, Sauthier P, Mayrand MH, Petignat P, Provencher D, Drouin P, et al. Human papillomavirus (HPV) DNA triage of women with atypical squamous cells of undetermined significance with Amplicor HPV and Hybrid Capture 2 assays for detection of high-grade lesions of the uterine cervix. J Clin Microbiol. janv 2011;49(1):48‑53.

107. Lapierre SG, Sauthier P, Mayrand MH, Dufresne S, Petignat P, Provencher D, et al. Human papillomavirus (HPV) DNA triage of women with atypical squamous cells of undetermined significance with cobas 4800 HPV and Hybrid Capture 2 tests for detection of high-grade lesions of the uterine cervix. J Clin Microbiol. avr 2012;50(4):1240‑4.

108. Ratnam S, Coutlee F, Fontaine D, Bentley J, Escott N, Ghatage P, et al. Aptima HPV E6/E7 mRNA test is as sensitive as Hybrid Capture 2 Assay but more specific at detecting cervical precancer and cancer. J Clin Microbiol. févr 2011;49(2):557‑64.

109. Pinto LA, Dillner J, Beddows S, Unger ER. Immunogenicity of HPV prophylactic vaccines: Serology assays and their use in HPV vaccine evaluation and development. Vaccine. 06 2018;36(32 Pt A):4792‑9.

110. Mehta PA, Sauter S, Zhang X, Davies SM, Wells SI, Myers KC, et al. Antibody response to human papillomavirus vaccination and natural exposure in individuals with Fanconi Anemia. Vaccine. 04 2017;35(48 Pt B):6712‑9.

111. Bissett SL, Wilkinson D, Tettmar KI, Jones N, Stanford E, Panicker G, et al. Human papillomavirus antibody reference reagents for use in postvaccination surveillance serology. Clin Vaccine Immunol CVI. mars 2012;19(3):449‑51.

Appendix A – Summary of the Canadian study QUEST: QUadrivalent Hpv vaccine Evaluation Study

Background:
A national study to comprehensively evaluate the durability of protection and efficacy of 2 doses of Q-HPV vaccine compared to 3 doses will be created by building on (1) the existing infrastructure of the extended dose Q-HPV provincial vaccine programmes in Quebec and British Columbia, (2) the existing provincial 3 dose programme in the Atlantic provinces and in Alberta and (3) the trial infrastructure and partnerships of a previous study, BCGov01 *A Controlled Trial to Assess the Immunogenicity of a Proposed Paediatric Dosing Schedule of Human Papillomavirus Vaccine* in Vancouver, Quebec City and Halifax.

Rationale:
Given the highly immunogenic response of the Q-HPV vaccine overall, high efficacy and the very high cost per dose, there has been broad research and programmatic interest in examining alternate and reduced dosing schedules for this vaccine. Studies have shown that antibody responses to HPV-16, 18, 6, 11 following a 2 dose pediatric regimen remained non-inferior, as compared to a 3-dose regimen in young adult women. These studies assessed antibody responses to a reduced dose Q-HPV regimen, but were not able to evaluate the clinical efficacy (i.e. prevention of cervical HPV infections or precancerous lesions) of the 2 versus 3 dose regimens. In addition there is still some uncertainty about whether immunogenicity evidence accurately correlates with efficacy/effectiveness. There is therefore a compelling need to examine the long-term efficacy of the 2 dose program in larger cohorts. QUEST proposes to do this in an economically and ethically efficient manner.

Objectives:

Primary Objective:

To evaluate if a 2-dose regimen of Q-HPV is non-inferior to a 3-dose schedule in the prevention of type specific persistent HPV16, 18, 6 or 11 infection in young women at 19/20 years of age.

Secondary Objectives:

- To evaluate if a 2-dose regimen of Q-HPV is non-inferior to a 3 dose schedule in the prevention of type specific persistent HPV16, 18, 6 or 11 infection at month 120 post dose 1 in girls vaccinated at the age of 9 – 12 years.
- To evaluate cumulative type specific persistence of HPV 16, 18, 6 or 11 at months 60, 84, 96 108 and 120 post dose 1 in girls vaccinated at the age of 9 – 12 years.
- To evaluate if a 2-dose regimen of Q-HPV is non-inferior to a 3 dose schedule in the prevention of self-reported anogenital warts in young women.
- To evaluate if a 2-dose regimen of Q-HPV is non-inferior to a 3 dose schedule in the prevention of type specific persistent HPV16, 18, 6 or 11 infection in young women at 15 years of age.
- To evaluate if a 2-dose regimen of Q-HPV is non-inferior to a 3 dose schedule in the prevention of type specific persistent HPV 31, 33, 35, 45, 52 and 58 infection in young women at 19 years of age
- To compare the mean antibody levels and seropositivity (for HPV types 16, 18, 6, or 11) in girls who have received 2 doses of Q-HPV to levels in girls who have received 3 doses at Months 60 and 120 post vaccination.

- To describe the trend over time of anti-HPV-16, -18, -6 -11 antibodies of those girls who took part in the BCGOV01 study up to month 36 after first immunization and who are now enrolled in this study, through to month 120.

Study Design:
This study is a longitudinal observational study with two cohorts of adolescent female volunteers. Cohort 1 will have received 2 doses of Q-HPV vaccine and Cohort 2 will have received 3 doses at the time of enrolment. Participants will be followed until they reach the age of 19, or when they are 120 months post dose 1, whichever comes last. The length of time for individual involvement will vary depending upon when the participant was enrolled post dose 1, age at dose 1 immunization and their age at enrolment. Participants will self-collect vaginal swabs or urine specimens every 6 months over a 5 year period, generally beginning in the year they turn 15 or at about 60 months post dose 1 if this time-point has already been achieved at study enrolment. These samples will be tested for the presence of HPV virus. Blood samples will be obtained on a sub-sample beginning in the year at which they reach 60 months post dose 1, and again 5 years later at about 120 months post dose 1 in a sub-sample of 700 enrolled participants. All vaginal swabs, urine specimens and blood samples will be sent to either the Provincial Health Service Laboratory located at the BC Centre for Disease Control (BCCDC) or the Laboratoire de Virologie Moléculaire, Centre de Recherche du Centre Hospitalier de l'Université de Montréal in Quebec or the Laboratoire de Santé Publique du Québec, for HPV antibody titers and genotyping. A health survey will be completed annually requesting demographic and age appropriate sexual health and sexual practises information. The survey will be completed on-line and stored on a secure database.

Population:
A total of 8666 Participants will be recruited in British Columbia (3612), Alberta (1445), Quebec (2167), and the Atlantic Provinces (1445). 4333 participants will be enrolled to each arm (2 doses of Q-HPV vaccine or 3 doses). Potential participants will be identified through various provincial means within regulatory and privacy boundaries.

Visit Schedule

This schedule for each individual participant will vary depending upon the age of the girl at enrolment and the length of time since the girl received dose 1 of the Q-HPV vaccine.

1. At study start, girls will be contacted according to accepted practice in each jurisdiction, and consent/assent will be sought as is applicable. An outline of expected study procedures will be provided. The outline of procedures will vary depending upon the year of dose one of HPV vaccine, the age at the time of the first vaccination and how long it has been since the first HPV vaccine at the time of study enrolment.
2. Once a year, while girls are in the study, the follow-up survey link will be emailed to them for survey completion (maximum of 11).
3. Vaginal swab or urine collecting will start in the year in which they reach age 15 or at about 60 months post dose 1 if this time-point has already been achieved at study enrolment. Once a girl starts sample collecting she will get a package to collect the swab or urine every 6mths for 5 years (total of 11).
4. If a girl is selected to take part in the blood collection she will be asked to do 2 samples. The first sample will be requested at about 60mths (5 years) after her first HPV vaccination and the second at about 10 years after her first HPV vaccination.

Study Management

The study will be managed centrally in BC with provinces recruiting participants at various enrolment sites in their respective provinces. Quebec will manage their participants separately (except for girls recruited from the BCGov01 Quebec City site who will be managed centrally), although the study procedures (questionnaires, HPV testing, serology) will be similar.

**Timeline**	Recruit and Enrol	August 2012 – Dec 2017
	First Visit First Subject	Aug 2012
	Follow-up	September 2012 - mid 2022
	Last Visit Last Subject	~ mid 2022

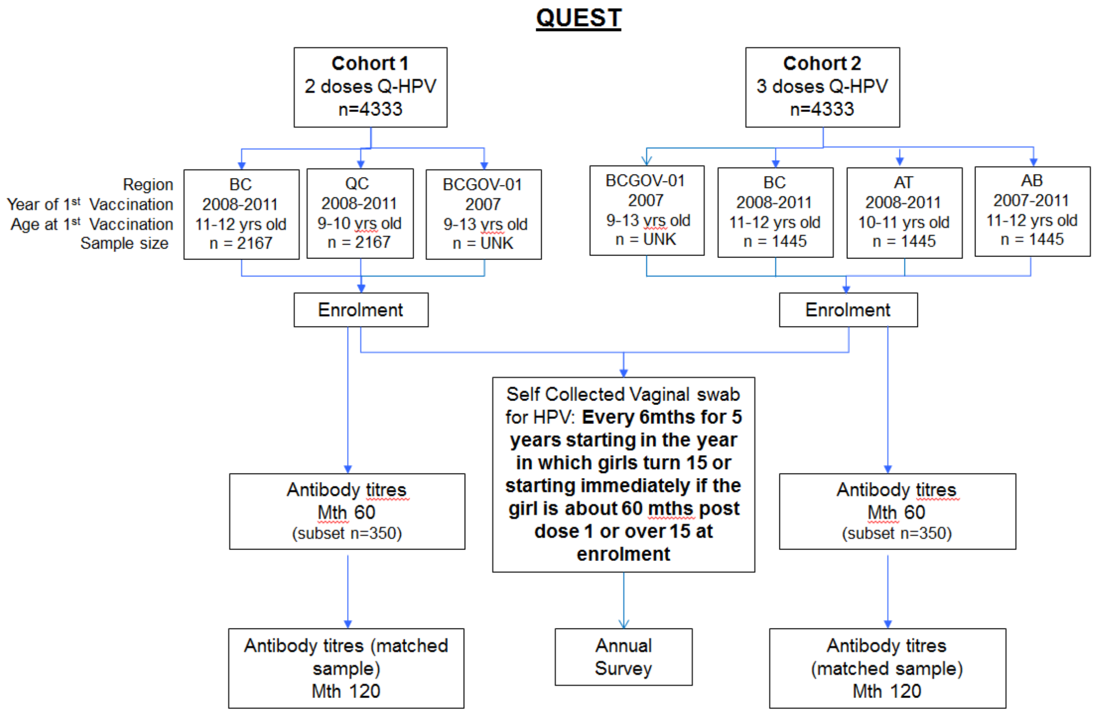

Appendix B – Conduct of the study

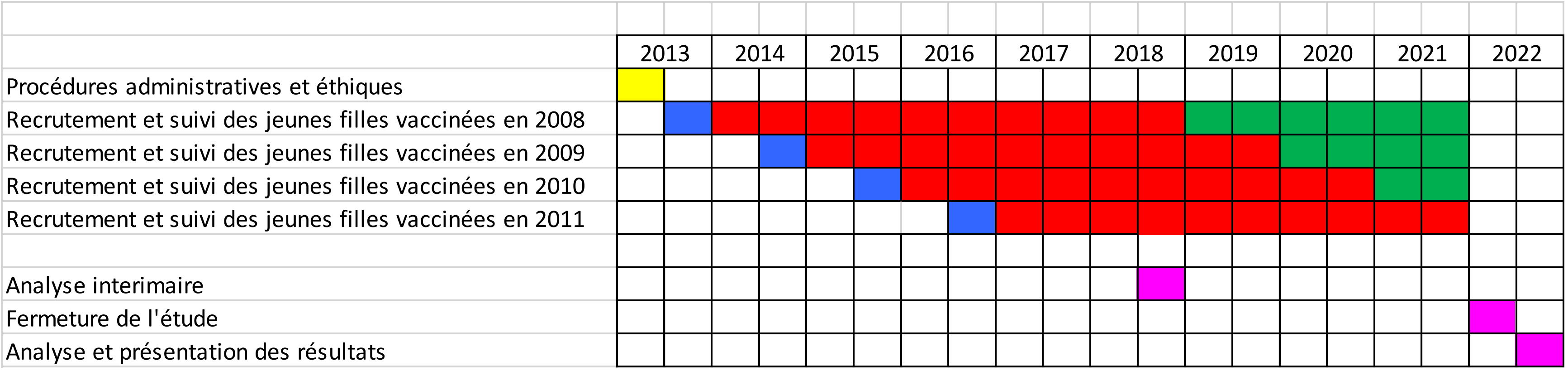

Supplement: Supplement 1. — Trial Protocol [file jamanetwopen-e2519095-s001.pdf]
