# Supplementary material for: Protection Against Persistent HPV-16/18 Infection After Different Number of Doses of Quadrivalent Vaccine in Girls and Young Women: A Randomized Clinical Trial
Source: JAMA Netw Open. 2025 Jul 8;8(7):e2519095. doi: 10.1001/jamanetworkopen.2025.19095 (PMC12238905; doi:10.1001/jamanetworkopen.2025.19095)
Supplement: Supplement 2. — eTable 1. Characteristics of Study Participants in the Annual Follow-Up Questionnaires eTable 2. HPV Positivity at Even-Numbered Time Points According to Vaccine Schedules eTable 3. Number of Swab Kits Sent, Received, and Tested at Each Even-Numbered Time Point for Up to 10 Years After the First Dose [file jamanetwopen-e2519095-s002.pdf]

Supplemental Online Content

Sauvageau C, Mayrand M-H, Ouakki M, et al. Protection against persistent HPV-16/18 infection after different doses of quadrivalent vaccine in girls and young women: a randomized clinical trial. *JAMA Netw Open*. 2025;8(7):e2519095. doi:10.1001/jamanetworkopen.2025.19095

eTable 1. Characteristics of Study Participants in the Annual Follow-Up Questionnaires

eTable 2. HPV Positivity at Even-Numbered Time Points According to Vaccine Schedules

eTable 3. Number of Swab Kits Sent, Received, and Tested at Each Even-Numbered Time Point for Up to 10 Years After the First Dose

This supplemental material has been provided by the authors to give readers additional information about their work.

© 2025 Sauvageau C et al. *JAMA Network Open*

eTable 1. Characteristics of study participants in the annual follow-up questionnaires.

**Characteristics, n (%)**	**2 doses (0, 6 months)**	**2+1 doses (0, 6, 60 months)**	**Total**
	**N=1,675**	**N=1,681**	**N=3,356**
**Tobacco**			
Ever smoked 1 cigarette	638 (38.1)	636 (37.8)	1274 (38.0)
**Sexual health**			
Had first menstruation	1673 (99.9)	1680 (99.9)	3353 (99.9)
Current user of hormonal contraception	1371 (81.9)	1362 (81.0)	2733 (81.4)
Ever had sex	1370 (81.8)	1410 (83.9)	2780 (82.8)
Ever had a STI[a]	205 (15.0)	208 (14.8)	413 (14.9)

STI, Sexually transmitted infection.
[a]Denominator includes only those who ever had sex.

eTable 2. HPV positivity at even-numbered time points according to vaccine schedules.

	**2 doses (0, 6 months) n = 8,510 (tests)**		**2+1 doses (0, 6, 60 months) n = 8,479 (tests)**	
	**n**	**%**	**n**	**%**
**High-risk HPV types**				
HPV 16	7	0.1	13	0.2
HPV 18	1	<0.1	2	<0.1
HPV 31	20	0.2	9	0.1
HPV 33	27	0.3	25	0.3
HPV 35	42	0.5	23	0.3
HPV 39	181	2.1	182	2.1
HPV 45	28	0.3	16	0.2
HPV 51	264	3.1	254	3.0
HPV 52	153	1.8	138	1.6
HPV 56	141	1.7	137	1.6
HPV 58	133	1.6	135	1.6
HPV 59	209	2.5	204	2.4
HPV 68	76	0.9	77	0.9
**Low-risk HPV types**				
HPV 6	0	0.0	4	<0.1
HPV 11	1	<0.1	3	<0.1
HPV 26	19	0.2	16	0.2
HPV 34	5	0.1	7	0.1
HPV 40	108	1.3	89	1.0
HPV 42	300	3.5	244	2.9
HPV 43	101	1.2	92	1.1
HPV 44	85	1.0	82	1.0
HPV 53	241	2.8	267	3.1
HPV 54	173	2.0	186	2.2
HPV 61	71	0.8	62	0.7
HPV 62	65	0.8	56	0.7
HPV 66	267	3.1	251	3.0
HPV 67	54	0.6	48	0.6
HPV 69	0	0.0	0	0.0
HPV 70	26	0.3	21	0.2
HPV 71	0	0.0	0	0.0
HPV 72	3	<0.1	2	<0.1
HPV 73	181	2.1	191	2.3
HPV 81	27	0.3	24	0.3
HPV 82	114	1.3	103	1.2
HPV 83	8	0.1	4	<0.1
HPV 84	150	1.8	135	1.6
HPV 89	148	1.7	141	1.7

Between-group comparisons were made using Fisher's exact test, with Bonferroni correction for multiple comparisons. All p-values were ≥0.05.

© 2025 Sauvageau C et al. *JAMA Network Open*

eTable 3. Number of swab kits sent, received, and tested at each even-numbered time point for up to 10 years after the first dose.

**Months since recruitment**	**6**		**18**		**30**		**42**		**54**		**Total No. (%)**	
**Years since 1st dose**	**6**		**7**		**8**		**9**		**10**			
**Group**	**2**	**2+1**	**2**	**2+1**	**2**	**2+1**	**2**	**2+1**	**2**	**2+1**	**2**	**2+1**
**Number randomized**	**1,675**	**1,681**	**1,675**	**1,681**	**1,675**	**1,681**	**1,675**	**1,681**	**1,675**	**1,681**	**1,675**	**1,681**
Lost to follow-up[a] (swab kits not sent)	0 (0.0)	0 (0.0)	21 (1.3)	21 (1.2)	32 (1.9)	33 (2.0)	51 (3.0)	49 (2.9)	68 (4.1)	61 (3.6)	68 (4.1)	61 (3.6)
COVID-19 interruption (swab kits not sent)	0 (0.0)	0 (0.0)	0 (0.0)	0 (0.0)	0 (0.0)	0 (0.0)	181 (10.8)	189 (11.2)	284 (17.0)	282 (16.8)	465 (27.8)	471 (28.0)
**Number of swab kits sent**	**1,675**	**1,681**	**1,654**	**1,660**	**1,643**	**1,648**	**1,443**	**1,443**	**1,323**	**1,338**	**7,738**	**7,770**
Swab kits not returned	29 (1.7)	22 (1.3)	42 (2.5)	48 (2.9)	109 (6.6)	109 (6.6)	157 (10.9)	173 (12.0)	141 (10.7)	158 (11.8)	478 (6.2)	510 (6.6)
**Number of swab kits returned**	**1,646**	**1,659**	**1,612**	**1,612**	**1,534**	**1,539**	**1,286**	**1,270**	**1,182**	**1,180**	**7,260**	**7,260**
Invalid samples	19 (1.2)	33 (2.0)	26 (1.6)	31 (1.9)	27 (1.8)	28 (1.8)	27 (2.1)	24 (1.9)	21 (1.8)	21 (1.8)	120 (1.7)	137 (1.9)
**Number of samples with result**	**1,627**	**1,626**	**1,586**	**1,581**	**1,507**	**1,511**	**1,259**	**1,246**	**1,161**	**1,159**	**7,140**	**7,123**

[a]Participants who indicated that they no longer wished to participate in the study or who had three consecutive incomplete procedures (i.e., swab not returned and/or questionnaire not completed).

© 2025 Sauvageau C et al. *JAMA Network Open*
