# Supplementary material for: Protection Against Persistent HPV-16/18 Infection After Different Number of Doses of Quadrivalent Vaccine in Girls and Young Women: A Randomized Clinical Trial
Source: JAMA Netw Open. 2025 Jul 8;8(7):e2519095. doi: 10.1001/jamanetworkopen.2025.19095 (PMC12238905; doi:10.1001/jamanetworkopen.2025.19095)
Supplement: Supplement 3. — Data Sharing Statement [file jamanetwopen-e2519095-s003.pdf]

Data Sharing Statement

Sauvageau. Protection Against Persistent HPV-16/18 Infection After Different Number of Doses of Quadrivalent Vaccine in Girls and Young Women. *JAMA Netw Open*. Published July 08, 2025. doi:10.1001/jamanetworkopen.2025.19095

Data
Additional Information: ClinicalTrials.gov, https://clinicaltrials.gov/study/NCT02009800?tab=table, Identifier: NCT02009800.
Data available: Yes
Data types: Data
How to access data: Data will be be made availble upon reasonable request to the corresponding author: chantal.sauvageau@inspq.qc.ca
When available: With publication

Supporting Documents
Document types: None

Additional Information
Who can access the data: Researchers whose proposed use of the data has been approved
Types of analyses: The data may be made available for analysis focusing on HPV prevention
Mechanisms of data availability: After approval of a proposal
